# Clinical outcomes and outcome measurement tools reported in randomised controlled trials of treatment for snakebite envenoming: A systematic review

**DOI:** 10.1371/journal.pntd.0009589

**Published:** 2021-08-02

**Authors:** Michael Abouyannis, Dinesh Aggarwal, David G. Lalloo, Nicholas R. Casewell, Mainga Hamaluba, Hanif Esmail

**Affiliations:** 1 Centre for Snakebite Research and Interventions, Liverpool School of Tropical Medicine, Liverpool, United Kingdom; 2 KEMRI-Wellcome Research Programme, Kilifi, Kenya; 3 Department of Medicine, University of Cambridge, Cambridge, United Kingdom; 4 Centre for Tropical Medicine & Global Health, Nuffield Department of Medicine, Oxford, United Kingdom; 5 MRC clinical trials unit at UCL, London, United Kingdom; 6 Institute for Global Health, University College London, London, United Kingdom; College of Health Sciences, Bayero University Kano, NIGERIA

## Abstract

**Background:**

Snakebite is a priority neglected tropical disease and causes a range of complications that vary depending on the snake species. Randomised clinical trials have used varied outcome measures that do not allow results to be compared or combined. In accordance with the Core Outcomes Measurements in Effectiveness Trials (COMET) initiative, this systematic review aims to support the development of a globally relevant core outcome set for snakebite.

**Methods:**

All randomised controlled trials, secondary analyses of randomised controlled trials and study protocols investigating the efficacy of therapeutics for human snakebite envenoming were eligible for inclusion. Study screening and data extraction were conducted in duplicate by two independent reviewers. All primary and secondary outcome measures were extracted and compiled, as were adverse event outcome measures. Similar outcome measures were grouped into domains. The study was prospectively registered with PROSPERO: CRD42020196160.

**Results:**

This systematic review included 43 randomised controlled trials, two secondary analyses and 13 study protocols. A total of 382 outcome measures were extracted and, after duplicates were merged, there were 153 unique outcomes. The most frequently used outcome domain (‘venom antigenaemia’) was included in less than one third of the studies. The unique outcomes were classified into 60 outcome domains. Patient-centred outcomes were used in only three of the studies.

**Discussion:**

Significant heterogeneity in outcome measures exists in snakebite clinical trials. Consensus is needed to select outcome measures that are valid, reliable, patient-centred and feasible. The results of this systematic review strongly support the development of a core outcome set for use in snakebite clinical trials.

## Author summary

Standardised outcome measures for snakebite randomised controlled trials are needed to enable results to be compared and combined between studies. This systematic review was conducted to understand the variations in outcome measure use in snakebite randomised controlled trials, and to create a comprehensive list of outcome measures from which to develop a core outcome set (COS). A total of 153 unique outcome measures were extracted from the 58 studies identified in this systematic review. Of these 153 unique outcome measures, 91 were used in only in a single study. Although a form of bedside whole blood clotting test was used in 30 of the 58 studies, 18 unique methods of measurement were identified. Only three studies, all conducted in the USA, included patient-centred outcomes. This systematic review demonstrates the strong need for a snakebite core outcome set, which will support the adoption of valid, reproducible, and patient-centred outcome measures, and enable downstream meta-analyses.

## Introduction

Global estimates indicate that there are 1·8 million envenomings and 94,000 deaths each year due to snakebite, with the highest burden in sub-Saharan Africa and Asia [1].

There is significant within-species and between-species variability in the toxins found in snake venoms [2], which account for the broad range of clinical manifestations caused by envenoming [3]. Syndromes of systemic envenoming include neurotoxicity, haemorrhage, and coagulopathy. Local effects can range from swelling to tissue necrosis, and are an important cause of disability and limb amputation [4]. Other effects of envenoming include myotoxicity, hypotension, and renal injury.

There has been limited funding for snakebite research, with a global average of under 5 million USD invested per annum [5], and a resulting paucity of clinical trials [6]. Antivenom is the only specific therapy for treating the aetiological toxins injected during snakebite, yet their use is rarely supported by clinical efficacy data or a rigorous regulatory framework [7]. However, in 2017 the World Health Organization reinstated snakebite envenoming as a category A neglected tropical disease [8], and thus funding to support snakebite management is anticipated to increase. Appropriate outcome measures are vital for ensuring that findings are relevant to patients and can appropriately inform policy makers. They need to be valid and reliable, particularly when surrogate endpoints are relied upon.

The Core Outcome Measures in Effectiveness Trials (COMET) Initiative has advocated for and supported the development of core outcome sets (COS) in clinical research [9]. These are developed by collaborative groups of researchers, clinicians, and patients; to identify an agreed minimum set of outcome measures for a disease area. By using a core outcome set, it is easier to compare, contrast and combine results of clinical trials, which has rarely been possible in the field of snakebite [10,11]. The first step toward developing a COS is to undertake a systematic review of the existing literature to inform. This systematic review aims to describe the heterogeneity in outcome measures used across clinical trials and will provide a comprehensive resource of outcome measures that can be considered when developing a COS.

## Methods

### Search strategy and selection criteria

Databases were searched for randomised controlled trials and trial protocols wherein therapeutics that inhibit venom, or its downstream pathological effects, were studied. MEDLINE, Cochrane CENTRAL, Web of Science and Embase were searched from database inception until the 23^rd^ of June 2020, with no language restriction, using the search terms ([“Snake bite” OR “snake envenomation” OR “snake venoms” OR “antivenoms” OR “antivenins] and [“randomised controlled trial” OR “randomised” OR “randomized” OR “randomly” OR “placebo” OR “double-blind” OR “single-blind” OR “clinical trial”]. Reference lists of included studies were searched. The following trial registries were searched: Australian trial register; International Standard Randomised Controlled Trial register; Clinical Trials Registry India; Chinese clinical trials registry; Clinical trial gov; Sri Lanka Clinical Trials Registry; Japan Primary Registries Network; WHO ICTRP. Full details of the search strategy were uploaded to PROSPERO (CRD42020196160).

Covidence systematic review software was used to compile, deduplicate and screen studies. Two reviewers (MA and DA) independently screened titles and abstracts, and subsequently the full-text articles. Full texts were translated to English language when necessary. Disagreements were resolved by consensus discussion with a third reviewer (HE). All reviewers (MA, DA and HE) are clinical academics with experience of interpreting clinical trials. Studies of adjunctive therapies that proposed to treat either antivenom hypersensitivity reactions or bite-site infection were excluded. Published secondary analyses of randomised controlled trials were included if they provided additional outcome measures.

### Data extraction

Prespecified data (as reported in S1 Text) were independently extracted and standardised by two authors (MA and DA). All primary, secondary, and adverse event outcome measures were extracted verbatim from full-text articles and study protocols. Outcome measures were grouped into the following predefined categories: haemorrhage; coagulopathy; neurotoxicity; local tissue damage; renal injury; cardiotoxicity; myotoxicity; mortality; venom antigenaemia; additional antivenom requirement; functional status; scoring system; composite outcome; or other.

### Data synthesis

After merging duplicate outcome measures, a data driven approach was used to classify them into domains. Each domain represented a grouping of outcomes that were deemed to be measuring a similar parameter. Consensus on domain allocations and domain names was reached by the primary authors. The characteristics of the studies and the outcome measures were summarised using descriptive statistics. The methodological quality of the primary and secondary outcome measures was assessed by the independent reviewers (MA and DA) using an established tool (as reported in S2 Text) [12]. The reviewers assessed whether each outcome measure was clearly stated; clearly defined; and patient-centred.

R version 4.0.3 was used for all analyses. The protocol was prospectively registered with PROSPERO (42020196160).

## Results

### Study screening

**Fig 1:**
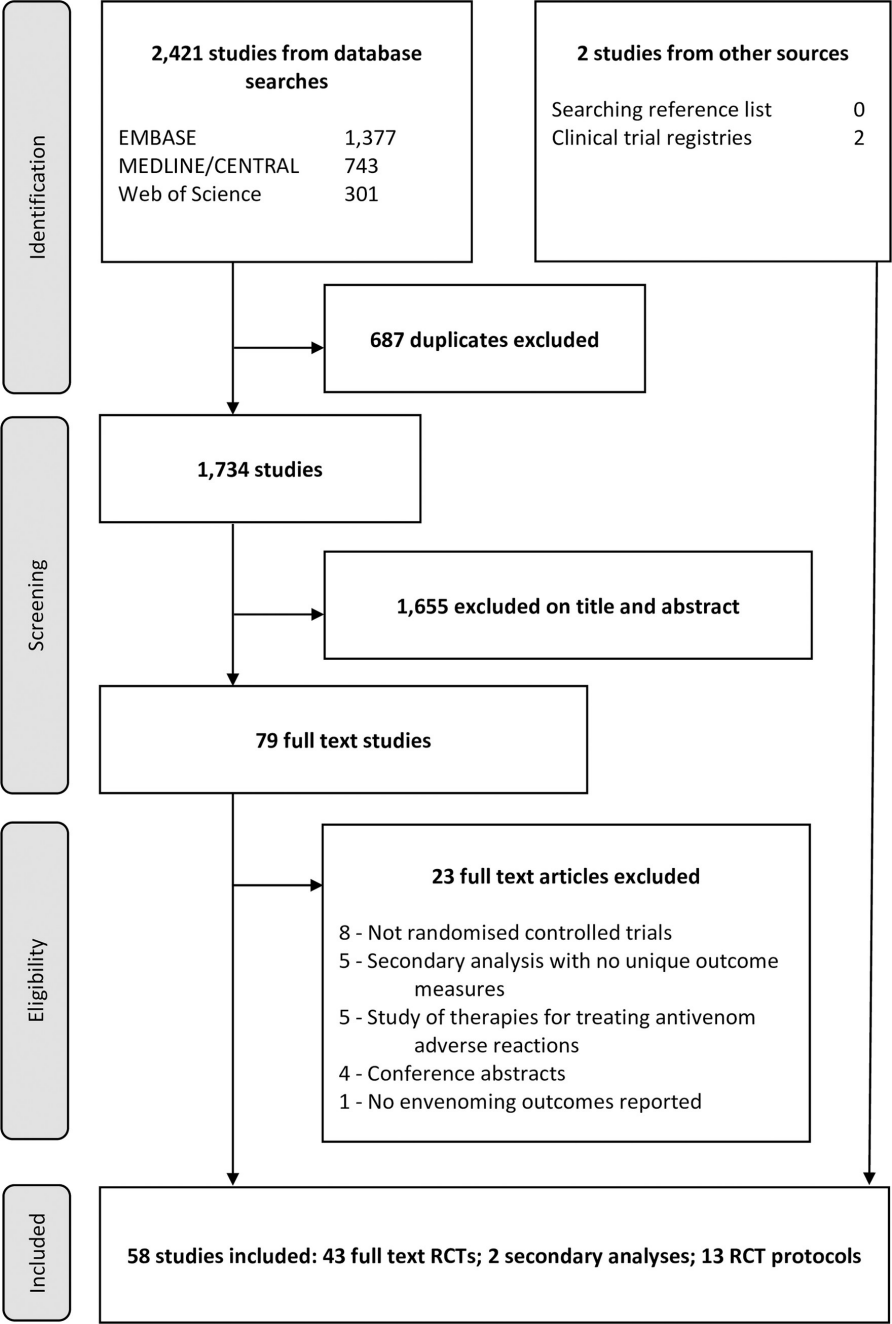
Study selection strategy. RCT = randomised controlled trial.

The database searches identified 2,421 studies, of which 687 were duplicates (Fig 1). Review of titles and abstracts identified 79 potentially eligible studies. All full texts were obtained and two required translation to English language. Searching of clinical trial registries identified two further protocols. Following full text review, 43 randomised controlled trials, 13 trial protocols and two published secondary analyses were included. Amongst the 13 included protocols, 6 had been terminated and 7 were ongoing. Two published secondary analyses [13,14] utilised data from the clinical trial published by Gerardo et al [15] and reported the additional outcome measures ‘opiate use’ and ‘the physical function domain of the SF-36 questionnaire’. Table 1 details the characteristics of all the included studies.

**Table 1:** Characteristics of randomised controlled trials and trial protocols.

Author (year)–protocol registration number	Country(s)	Blinding	N	Snake species	Syndrome of envenoming	Comparison	Products	Primary outcome measure* (verbatim)^†^	Primary outcome measure category*	Primary outcome clearly defined*^‡^ (yes/no)?	Primary outcome patient-centred* (yes/no)?	Adverse event outcome domains* (clearly defined^‡^–yes/no?)
Randomised controlled trials												
Isbister et al. (2013) [24]—ACTRN12607000620426	Australia	Open label	65	*Pseudonaja textilis*, *Notechis scutatus*, *Tropidechis carinatus* and *Hoplocephalus* spp.	Coagulopathy, haemorrhage and local tissue damage	FFP vs standard of care	Fresh frozen plasma	Proportion with an INR<2 at six hours post antivenom	Coagulopathy	Yes	No	Anaphylaxis (yes)
Mendonca-da-Silva et al. (2017) [62]—ISRCTN12845255	Brazil	Open label	116	*Bothrops Lachesis* and *Crotalus* spp.	Coagulopathy, haemorrhage and local tissue damage	Different antivenom products	Lyophilised Polyvalent Bothrops-Lachesis-Crotalus (Instituto Butantan) Liquid fill polyvalent antivenoms: *Bothrops* or *Bothrops-Lachesis* or *Bothrops-Crotalus* antivenom (Instituto Butantan/Instituto Vital Brazil/FUNED)	NA	NA	NA	NA	Anaphylaxis (yes); serum sickness (no)
de Oliveira Pardal et al. (2004) [52]	Brazil	Open label	74	*Bothrops atrox*, *Lachesis muta* and *Bothrops marajoensis*	Coagulopathy, haemorrhage and local tissue damage	Different antivenom products	Antibotropico-laquetico polyvalent *Bothrops-Lachesis* antivenom (Instituto Butantan) Antibotropico polyvalent *Bothrops* whole IgG antivenom (FUNED)	NA	NA	NA	NA	Non-specific early reaction (no)
Jorge et al. (1995) [63]	Brazil	Double blind	170	*Bothrops* spp.	Coagulopathy, haemorrhage and local tissue damage	Different doses of antivenom	Antibotropico polyvalent Bothrops antivenom (Instituto Butantan)	NA	NA	NA	NA	NA
Cardoso et al. (1993) [54]	Brazil	Double blind	121	*Bothrops* spp.	Coagulopathy, haemorrhage and local tissue damage	Different antivenom products	Antibotropico polyvalent Bothrops antivenom (Instituto Butantan) Antibotropico polyvalent Bothrops antivenom (Instituto Vital Brazil) Antibotropico polyvalent Bothrops whole IgG antivenom (FUNED)	NA	NA	NA	NA	Serum sickness (no); non-specific early reaction (no)
Theakston et al. (1992) [84]	Brazil	Open label	118	*Bothrops* spp.	Coagulopathy, haemorrhage and local tissue damage	Different antivenom products	Antibotropico polyvalent Bothrops antivenom (Instituto Butantan) Antibotropico polyvalent Bothrops antivenom (Instituto Vital Brazil) Antibotropico polyvalent Bothrops whole IgG antivenom (FUNED)	NA	NA	NA	NA	NA
Miao et al. (2003) [28]	China	Open label	46	Not identified	Coagulopathy, haemorrhage and local tissue damage	Qingwen Baidu Decoction vs standard of care	Qingwen Baidu Decoction "anti-cobra serum" "anti-viper serum" "anti-acutus serum"	NA	NA	NA	NA	NA
Otero-Patino et al. (2012) [53]	Colombia	Double blind	72	*Bothrops asper*	Coagulopathy, haemorrhage and local tissue damage	Different antivenom products	Polyvalent Bothrops-Lachesis-Crotalus F(ab’)2 antivenom (ICP) [pepsin digestion caprylic acid fractionation] Polyvalent Bothrops-Lachesis-Crotalus whole IgG antivenom (ICP) [caprylic acid fractionation]	NA	NA	NA	NA	Anaphylaxis (yes); early hypersensitivity reaction (yes); serum sickness (no)
Otero et al. (2006) [70]	Colombia	Double blind	67	*Bothrops asper*	Coagulopathy, haemorrhage and local tissue damage	Different antivenom products	Polyvalent Bothrops-Lachesis-Crotalus whole IgG antivenom (ICP) [ammonium sulfate precipitation and pepsin digestion] Polyvalent Bothrops-Lachesis-Crotalus whole IgG antivenom (ICP) [caprylic acid fractionation] Monovalent Bothrops atrox whole IgG antivenom (Instituto Nacional de Salud)	Cessation of local and systemic haemorrhage within the first 6 h of treatment and permanent recovery of blood coagulability no later than 6h after the onset of serotherapy were considered as the criteria of efficacy of the initial antivenom dose during phase II of the study. If these criteria were not fulfilled, an additional dose of three vials of the same antivenom was administered.	Composite outcome	No	No	Non-specific early reaction (no)
Otero et al. (1999) [65]	Colombia	Double blind	53	*Bothrops* spp. and *Porthidium* spp.	Coagulopathy, haemorrhage and local tissue damage	Different antivenom products	Polyvalent Borthrops-Lachesis-Crotalus whole IgG antivenom (ICP) Monovalent Bothrops atrox whole IgG antivenom (ICP)	NA	NA	NA	NA	Serum sickness (no); early hypersensitivity reaction (yes)
Otero-Patino et al. (1998) [64]	Colombia	Double blind	79	*Bothrops* spp.	Coagulopathy, haemorrhage and local tissue damage	Different antivenom products	Polyvalent Bothrops F(ab’)2 antivenom (Instituto Butantan) Polyvalent Bothrops whole IgG antivenom (Instituto Butantan) Monovalent Bothrops atrox whole IgG antivenom (Instituto Nacional de Salud)	The cessation of local and systemic bleeding and no progression of swelling between six and 12 hr after the initial dose of antivenom was considered an adequate therapeutic response	Composite outcome	No	No	Serum sickness (no); non-specific early reaction (no)
Otero et al. (1996) [67]	Colombia	Double blind	39	*Bothrops atrox*	Coagulopathy, haemorrhage and local tissue damage	Different antivenom products	Polyvalent Borthrops-Lachesis-Crotalus antivenom (ICP) Monovalent Bothrops atrox whole IgG antivenom (ICP)	NA	NA	NA	NA	Anaphylaxis (no); non-specific early reaction (no)
Smalligan et al. (2004) [69]	Ecuador	Double blind	210	*Bothrops spp*. and *Lachesis muta*	Coagulopathy, haemorrhage and local tissue damage	Different antivenom products	Polyvalent Bothrops antivenom whole IgG (Instituto Nacional de Higiene y Medicina Tropical “Leopoldo Izquieta Perez”) Polyvalent Bothrops antivenom (Instituto Butantan) Polyvalent Bothrops-Lachesis-Crotalus whole IgG antivenom (Instituto Nacional de Salud)	Permanent restoration of blood coagulability after 6 hours and 24 hours (20WBCT)	Coagulopathy	Yes	No	Non-specific early reaction (no)
Sagar et al. (2020) [34]—CTRI/2015/05/005826	India	Open label	140	Not identified	Coagulopathy, haemorrhage, local tissue damage and renal injury	Different doses of antivenom	Polyvalent F(ab’)2 antivenom (VINS)	Number developing AKI or mortality (AKI as per KDIGO criteria)	Composite outcome	Yes	No	Anaphylaxis (no); non-specific early reaction (no)
Sarin et al. (2017) [32]—CTRI/2015/06/005893	India	Open label	51	Not identified	Neurotoxicity	Different doses of antivenom	Polyvalent F(ab’)2 antivenom (VINS)	Duration of mechanical ventilation (from time of intubation to time of extubation)—as per study protocol	Neurotoxicity	Yes	No	Non-specific early reaction
Paul et al. (2007) [19]	India	Open label	80	Not identified	Coagulopathy, haemorrhage, local tissue damage and renal injury	Heparin vs standard of care	Dalteparin Polyvalent Fab2 antivenom (Serum Institute of India)	NA	NA	NA	NA	NA
Paul et al. (2004) [38]	India	Double blind	100	Not identified	Coagulopathy, neurotoxicity and renal injury	Different doses of antivenom	Polyvalent Fab2 antivenom (Serum Institute of India)	NA	NA	NA	NA	NA
Srimannarayana et al. (2004) [66]	India	Open label	60	Not identified	Coagulopathy and renal injury	Different doses of antivenom	Polyvalent Fab2 antivenom (Serum Institute of India)	NA	NA	NA	NA	Non-specific early reaction (no)
Paul et al. (2003) [22]	India	Open label	122	Not identified	Coagulopathy and renal injury	Heparin vs standard of care	Heparin infusion Polyvalent Fab2 antivenom (Serum Institute of India)	NA	NA	NA	NA	NA
Tariang et al. (1999) [39]	India	Single blind	60	Not identified	Coagulopathy, local tissue damage, neurotoxicity and renal injury	Different doses of antivenom	Polyvalent Fab2 antivenom (Serum Institute of India)	Normalisation of coagulopathic (Clotting time more than 15 minutes) or neurotoxic (confusion, bilateral ptosis, dysarthria, dysphonia, respiratory distress, respiratory paralysis, muscle weakness, diplopia, bradycardia, hypotension) parameters or death	Composite outcome	No	No	NA
Thomas et al. (1985) [37]	India	Open label	53	Not identified	Coagulopathy local tissue damage and renal injury	Different doses of antivenom	Polyvalent Fab2 antivenom (Haffkine Biopharmaceutical Corporation)	NA	NA	NA	NA	NA
Reid et al. (1963)[16]	Malaysia	Double blind	100	*Calloselasma rhodostoma*	Coagulopathy, haemorrhage and local tissue damage	Antivenom vs placebo vs steroids	“Agkistrodon rhodostoma” antivenom (Queen Saovabha Institute) Prednisolone	NA	NA	NA	NA	Anaphylaxis (no); serum sickness (no)
Tin et al. (1992) [20]	Myanmar	Open label	20	*Daboia russelii*	Coagulopathy and renal injury	Heparin vs placebo	Heparin infusion Monovalent Russell’s viper antivenom (Myanmar Pharmaceutical Industry)	NA	NA	NA	NA	NA
Myint-Lwin et al. (1989) [21]	Myanmar	Open label	28	*Daboia russelii*	Coagulopathy and renal injury	Heparin vs placebo	Heparin infusion Monovalent Russell’s viper antivenom (Burma Pharmaceutical Industry)	NA	NA	NA	NA	NA
Alirol et al. (2017) [48]- NCT01284855	Nepal	Double blind	155	Not identified	Neurotoxicity	Different doses of antivenom	Polyvalent F(ab’)2 antivenom (VINS)	In-hospital death OR the need for assisted ventilation (clinical indications for intubation and assisted ventilation were (1) absent gag reflex, (2) presence of paradoxical breathing, (3) respiratory distress or cyanosis, whichever was detected first, and/or (4) oxygen saturation <90% despite high flow oxygen supplementation) OR worsening or recurrence of neurotoxicity (defined as the appearance of 2 new neurotoxic signs OR the appearance of a severe neurotoxic sign) after the initial dose of antivenom	Composite outcome	Yes	No	Anaphylaxis (no); full adverse event reporting (yes)
Abubakar et al. (2010) [56]—ISRCTN01257358	Nigeria	double blinded	400	*Echis ocellatus*	Coagulopathy, haemorrhage and local tissue damage	Different antivenom products	EchiTAb Plus-ICP polyvalent whole IgG antivenom (ICP) EchiTAb G monovalent E ocellatus whole IgG antivenom (MicroPharm)	Permanent restoration of blood coagulability, judged by 20WBCT at 6 hours after initiation of antivenom treatment. Permanent implied restoration after which there was no (further) recurrence of blood incoagulability. This was assessed by repeating the 20WBCT 6, 12, 18, 24 and 48 hr after the initial dose of antivenom.	Coagulopathy	Yes	No	Anaphylaxis (yes); pyrogenic reaction (yes); serum sickness (no)
Meyer et al. (1997) [68]	Nigeria	Open label	39	*Echis ocellatus*	Coagulopathy, haemorrhage and local tissue damage	Different antivenom products	Polyvalent IPSER Africa F(ab’)2 antivenom (Pasteur Mérieux Serum) EchiTab monovalent *E*. *ocellatus* Fab antivenom (Therapeutic Antibodies Ltd)	NA	NA	NA	NA	Anaphylaxis (yes)
Warrell et al. (1980) [57]	Nigeria	Open label	14	*Echis ocellatus*	Coagulopathy, haemorrhage and local tissue damage	Different antivenom products	North and West African Bitis-Echis-Naja polyvalent antivenom (Behringwerke) Monovalent *Echis ocellatus* antivenom (Pasteur Paris)	NA	NA	NA	NA	Non-specific early reaction (no)
Warrell et al. (1974) [58]	Nigeria	Open label	46	*Echis ocellatus*	Coagulopathy, haemorrhage and local tissue damage	Different antivenom products	North and West African *Bitis-Echis-Naja* polyvalent antivenom (Behringwerke) Monovalent *Echis* antivenom (SAIMR)	NA	NA	NA	NA	Non-specific early reaction (no)
Qureshi et al. (2013) [71]	Pakistan	Double blind	74	Not identified	Coagulopathy and local tissue damage	Different antivenom products	Polyvalent Pakistani antivenom (National Institute of Health, Islamabad) Polyvalent F(ab’)2 antivenom (VINS)	NA	NA	NA	NA	Early hypersensitivity reaction (yes)
Trevett et al. (1995) [26]	Papua New Guinea	Double blind	50	*Oxyuranus scutellatus canni*	Neurotoxicity	Edrophonium vs Amifampridine vs placebo (all groups received atropine)	Edrophonium Amifampridine Atropine	NA	NA	NA	NA	Non-specific early reaction (no)
Watt et al. (1989) [46]	Philippines	Double blind	8	*Naja philippinensis*	Neurotoxicity	Different doses of antivenom	Monovalent Philippine cobra antivenom (Philippine MOH) All groups received edrophonium and atropine	NA	NA	NA	NA	Non-specific early reaction (no); serum sickness (no)
Watt et al. (1986) [25]	Philippines	Double blind	10	*Naja philippinensis*	Neurotoxicity	Atropine and Edrophonium vs Placebo	Atropine and Edrophonium	Number of seconds that upper lid retraction could be maintained during upward gaze and the proportion of the iris uncovered during maximal effort to open the eyes	Neurotoxicity	Yes	No	NA
Isbister et al. (2017) [31]—SLCTR/2010/011	Sri Lanka	Open label	141	*Daboia russelii*	Coagulopathy, haemorrhage and renal injury	Fresh frozen plasma vs standard of care	Fresh frozen plasma Polyvalent F(ab’)2 antivenom (VINS)	The primary outcome was the proportion of patients with an INR of <2 at 6 h post-antivenom administration	Coagulopathy	Yes	No	Anaphylaxis (yes); Transfusion-related acute lung injury (yes)
Ariaratnam et al. (2001) [47]	Sri Lanka	Open label	43	*Daboia russelii*	Coagulopathy, local tissue damage, myotoxicity, neurotoxicity and renal injury	Different antivenom products	Monovalent Sri Lankan Daboia russelii Fab antivenom (Protherics) Polyvalent Fab2 antivenom (Haffkine Biopharmaceutical Corporation)	NA	NA	NA	NA	Anaphylaxis (no); early hypersensitivity reaction (yes); non-specific early reaction (no); pyrogenic reaction (no)
Sellahewa et al. (1995) [17]	Sri Lanka	Single blind	63	*Hypnale hypnale*	Local tissue damage	Antivenom vs placebo	Polyvalent Fab2 antivenom (Haffkine Biopharmaceutical Corporation)	NA	NA	NA	NA	Early hypersensitivity reaction (no)
Sellahewa et al. (1994) [27]	Sri Lanka	Open label	15	*Daboia russelii* and *Hypnale hypnale*	Coagulopathy, local tissue damage and neurotoxicity	IVIG vs standard of care	Intravenous immunoglobulin (IVIG) (Sandoglobulin, Sandoz Pharmaceuticals) SII Polyvalent ASV IP Fab2 antivenom (Serum Institute of India)	NA	NA	NA	NA	Anaphylaxis (no)
Rojnuckarin et al. (2006) [18]	Thailand	Double blind	28	*Trimeresurus macrops*, *Trimeresurus albolabris* and *Trimeresurus macrops*	Local tissue damage	Antivenom vs placebo	Polyvalent Green pit viper F(ab’)2 antivenom (QSMI, Thai Red Cross)	The degree of oedema is defined by the differences in circumferences of the affected and unaffected sides of limbs, measured (in cm) at the same distances from nearby joints. The points with the maximal differences were chosen and marked for the next measurements. The main outcome is the percentage reduction in limb circumference, calculated as the reduction in limb circumference on each day after intervention, divided by the initial limb circumference, and multiplied by 100.	Local tissue damage	Yes	No	NA
Warrell et al. (1986) [59]	Thailand	Open label	46	*Calloselasma rhodostoma*	Coagulopathy, haemorrhage and local tissue damage	Different antivenom products	Monovalent Malayan pit viper antivenom (QSMI, Thai Red Cross) Monovalent Malayan pit viper antivenom (Thai Government Pharmaceutical Organization) Monovalent Malayan pit viper antivenom (Twyford Pharmaceutical)	NA	NA	NA	NA	Serum sickness (no); non-specific early reaction (no); pyrogenic reaction (yes)
Gerardo et al. (2017) [15]—NCT01864200	USA	Double blind	76	*Agkistrodon contortrix*	Local tissue damage	Antivenom vs placebo	CroFab Polyvalent Crotalid Fab antivenom (Protherics)	Limb function 14 days after envenomation, measured by the Patient-Specific Functional Scale	Functional status	Yes	Yes	Full adverse event reporting (yes)
Gerardo et al. (2019) [13]–secondary analysis of NCT01864200	^	^	^	^	^	^	^	NA	NA	NA	NA	NA
Freiermuth et al. (2018) [14]—secondary analysis of NCT01864200	^	^	^	^	^	^	^	NA	NA	NA	NA	NA
Bush et al. (2015) [85]—NCT00636116	USA	Double blind	123	*Crotalinae*	Coagulopathy	Different antivenom products	Anavip polyvalent Crotalid F(ab’)2 antivenom (Instituto Bioclon S.A.) CroFab Polyvalent Crotalid Fab antivenom (Protherics)	Coagulopathy between the end of maintenance dosing and study day 8 (+/- 1 day). Coagulopathy was defined as platelet count less than 150,000/mm3, fibrinogen less than 150 mg/dL, or use of antivenom to treat a coagulation abnormality between the end of maintenance dosing and study day 5	Composite outcome	Yes	No	Full adverse event reporting (yes); non-specific early reaction (no); serum sickness (no)
Boyer et al. (2013) [86]—NCT00868309	USA	Open label	12	*Crotalinae*	Coagulopathy	Different antivenom products	CroFab Polyvalent Crotalid Fab antivenom (Protherics) Anavip polyvalent Crotalid F(ab’)2 antivenom (Instituto Bioclon S.A.)	Detection of plasma venom levels during the post-acute treatment period	Venom antigenaemia	No	No	Anaphylaxis (no); full adverse event reporting (yes); serum sickness (no)
Dart et al. (2001) [42]	USA	Open label	31	*Crotalinae* (excluding *Agkistrodon contortrix*)	Coagulopathy and local tissue damage	Different doses of antivenom	CroFab Polyvalent Crotalid Fab antivenom (Protherics)	Snakebite severity score[41]	Scoring system	Yes	No	Serum sickness (yes); non-specific early reaction (no)
Protocols of active studies												
Isbister et al. (2011) [43]—ACTRN12611000588998	Australia	Double blind	-	*Pseudechis porphyriacus*	Coagulopathy and myotoxicity	Antivenom vs placebo	Monovalent Tiger Snake F(ab’)2 antivenom (Commonwealth Serum Laboratories) Placebo	The proportion of patients with myotoxicity defined as a peak creatine kinase greater than 1000U/L.	Myotoxicity	Yes	No	Early hypersensitivity reaction (yes); serum sickness (yes)
Ghorbani et al. (2013) [82]—IRCT2012122411873N1	Iran	Double blind	-	Not reported	Local tissue damage	Steroid vs placebo	Dexamethasone infusion	Limb oedema (volume of limb according to shift of water in a dish scale in mL)	Local tissue damage	Yes	No	Full adverse event reporting (no)
Mousavi et al. (2020) [30]—IRCT20180515039672N2	Iran	Double blind	-	Not identified	Coagulopathy, local tissue damage and neurotoxicity	Different antivenom products	SnaFab polyvalent antivenom (Padra Serum) Polyvalent Snake Antivenin (Razi Serum and Vaccine Research Institute)	Percentage of victims with improving in snakebite symptoms (A) Stopping progression of swelling B) Normalized coagulation abnormalities C) Stopping the progression of neurotoxicity)	Other	No	No	NA
Lamb et al. (2020) [44]—NCT04210141	Myanmar	Open label	-	*Daboia siamensis*	Coagulopathy and renal injury	Different doses of antivenom	Monospecific lyophilized F(ab)’2 viper antivenom (Burma Pharmaceutical Industry)	Blood coagulation at 6 hours as measured by the 20-minute WBCT (binary outcome)	Coagulopathy	Yes	No	Anaphylaxis (yes); full adverse event reporting (yes)
Jensen et al. (2012) [87]—ACTRN12612001062819	Papua New Guinea	Double blind	-	*Oxyuranus scutellatus*	Coagulopathy and neurotoxicity	Different antivenom products	Monovalent Papuan taipan F(ab’)2 antivenom (ICP) Monovalent Taipan F(ab’)2 antivenom (Commonwealth Serum Laboratories)	Prevention of airway obstruction or respiratory failure post-antivenom	Neurotoxicity	Yes	No	NA
Gawarammana et al. (2016) [29]—SLCTR/2016/012	Sri Lanka	Double blind	-	*Daboia russelii* and *Echis carnatus*	Coagulopathy, local tissue damage, myotoxicity, neurotoxicity and renal injury	Different antivenom products	Polyvalent Sri Lankan antivenom (ICP) Polyvalent F(ab’)2 antivenom (VINS)	1) Early anaphylactic-like reactions (up to 04 hrs) i. Mild: pruritus and/or urticarial only ii. Severe: Gastrointestinal symptoms (vomiting, diarrhoea, colicky abdominal pain) iii. Bronchospasm, or fall in systolic blood pressure below 90 mmHg 2) Early Pyrogenic reactions (up to 4 hours): increase oral temperature 38°C or above with or without rigors 3) Late serum sickness type antivenom reactions (2 weeks later): urticarial, pruritus, arthralgia, fever	Other	Yes	No	NA
Kularatne et al. (2010) [33]—SLCTR/2010/006	Sri Lanka	Open label	-	*Bungarus Caeruleus*	Neurotoxicity	Different doses of antivenom	Polyvalent F(ab’)2 antivenom (VINS)	The duration of mechanical ventilation (time in hours from the onset of intubation to extubation)	Neurotoxicity	Yes	No	NA
Protocols of terminated studies												
Grais et al. (2016) [88]—NCT02694952	Central African Republic	Double blind	-	Not reported	Coagulopathy and haemorrhage	Different antivenom products	EchiTAb-Plus polyvalent whole IgG antivenom (ICP) FAV Afrique polyvalent F(ab’)2 antivenom (Sanofi Pasteur)	Number of patients needing a third dose of antivenom, needing a blood transfusion, or dying [TimeFrame:28 days after enrolment]	Composite outcome	Yes	No	Full adverse event reporting (no)
Isbister et al. (2015) [36]—ACTRN12615000264583	Australia	Open label	-	*Notechis scutatus* and *Pseudonaja textilis*	Coagulopathy, haemorrhage, local tissue damage, myotoxicity and renal injury	Early administration vs standard administration of antivenom	Monovalent Tiger Snake F(ab’)2 antivenom (Commonwealth Serum Laboratories) Brown Snake Antivenom (Commonwealth Serum Laboratories)	Proportion of patients with significant envenomation defined as the development of one or more of the following effects (composite outcome): i) Myotoxicity defined as a peak CK greater than 1000U/L and local or systemic myalgia ii) Neurotoxicity: paralysis of 2 or more muscle groups (extra-ocular + bulbar) or respiratory paralysis iii) Major bleeding: defined by the International Society on Thrombosis and Haemostasis as fatal bleeding, symptomatic bleeding in a critical organ (e.g. intracranial haemorrhage) or bleeding resulting in a drop of haemoglobin >20g/L or requiring blood transfusion iv) Acute kidney failure injury: defined by the RIFLE criteria (creatinine increasing by 2x or more; <0·5ml/kg/hr urine output over12h)	Composite outcome	Yes	No	Anaphylaxis (yes)
Krishnan et al. (2016) [35]—CTRI/2016/10/007360	India	Double blind	-	Not identified	Haemorrhage, local tissue damage, neurotoxicity and renal injury	N-Acetyl Cysteine vs placebo	N-Acetyl Cysteine Antivenom product not specified	To compare the incidence and severity of acute kidney injury.	Renal injury	No	No	Non-specific early reaction (no)
Garcia et al. (2008) [89]—NCT00639951	Mexico	Open label	-	Not identified	Coagulopathy, local tissue damage and myotoxicity	Different doses of antivenom	Antivipmyn Polyvalent Crotalinae F(ab’)2 antivenom (Instituto Bioclon)	Resolution of systemic signs and symptoms of snake bite envenomation expressed as percentage of patients requiring additional antivenom and percentage of patients that are stable.	Additional antivenom requirement	No	No	NA
Isbister et al. (2008) [23]—ACTRN12608000611325	Sri Lanka	Open label	-	*Daboia russelii*	Coagulopathy and haemorrhage	FFP vs placebo	Fresh frozen plasma Polyvalent F(ab’)2 antivenom (VINS) Polyvalent Fab2 antivenom (Haffkine Biopharmaceutical Corporation)	The proportion of patients with a significant return of coagulation function defined by an International Normalised Ratio (INR) < 2.0 (or prothrombin time (PT) < 24 seconds where INR was not performed)	Coagulopathy	Yes	No	Anaphylaxis (yes); Transfusion-related acute lung injury (yes)
Kerns et al. (2006) [40]—NCT00303303	USA	Double blind	-	*Agkistrodon contortrix*	Coagulopathy and local tissue damage	Different doses of antivenom	CroFab Polyvalent Crotalid Fab antivenom (Protherics)	NA	NA	NA	NA	NA

Studies are ordered alphabetically by country within each of the following groups: published randomised controlled trials; protocols of active, incomplete or terminated randomised controlled trials; and protocols of terminated randomised controlled trials.

Published secondary analyses are displayed below the corresponding primary randomised controlled trial.

Snake species is reported to species, genus or sub-family taxonomic rank, depending on the level of identification in each study.

Syndrome of envenoming is reported based on the study inclusion criteria and the descriptions of participant characteristics within each study.

20WBCT = 20-minute whole blood clotting test, AKI = acute kidney injury, CK = creatinine kinase, Fab = antigen-binding fragment, FFP = fresh frozen plasma, FUNED = Fundação Ezequiel Dias, ICP = Instituto Clodomiro Picado, Ig-immunoglobulin, INR = international normalised ratio, IVIG = intravenous immunoglobulin, KDIGO = Kidney Disease: Improving Global Outcomes, MOH = Ministry of Health, N = the number of participants randomised, PT = prothrombin time, RCT = randomised controlled trial, and SAIMR = South African Institute for Medical Research.

* For studies that did not clearly report a primary outcome measure, or adverse events, ‘NA’ has been listed in the table to highlight that this was not available.

^†^ Where necessary, for the purposes of clarity, minor changes to the wording of outcome measures have been made. For example, in certain studies the outcome measure was partly defined in the methods section and partly defined in the results section, and thus required joining.

^‡^ ‘Clearly defined’ was defined as being reproducible by another researcher, including clear descriptions of time points, the person measuring the outcome, how the outcome was measured, and where the outcome was measured. ‘Patient-centred outcomes’ were defined as a measure of what the participant can do or how they feel, such as activities of daily living or ability to work.

### Characteristics of included randomised controlled trials

**Fig 2:**
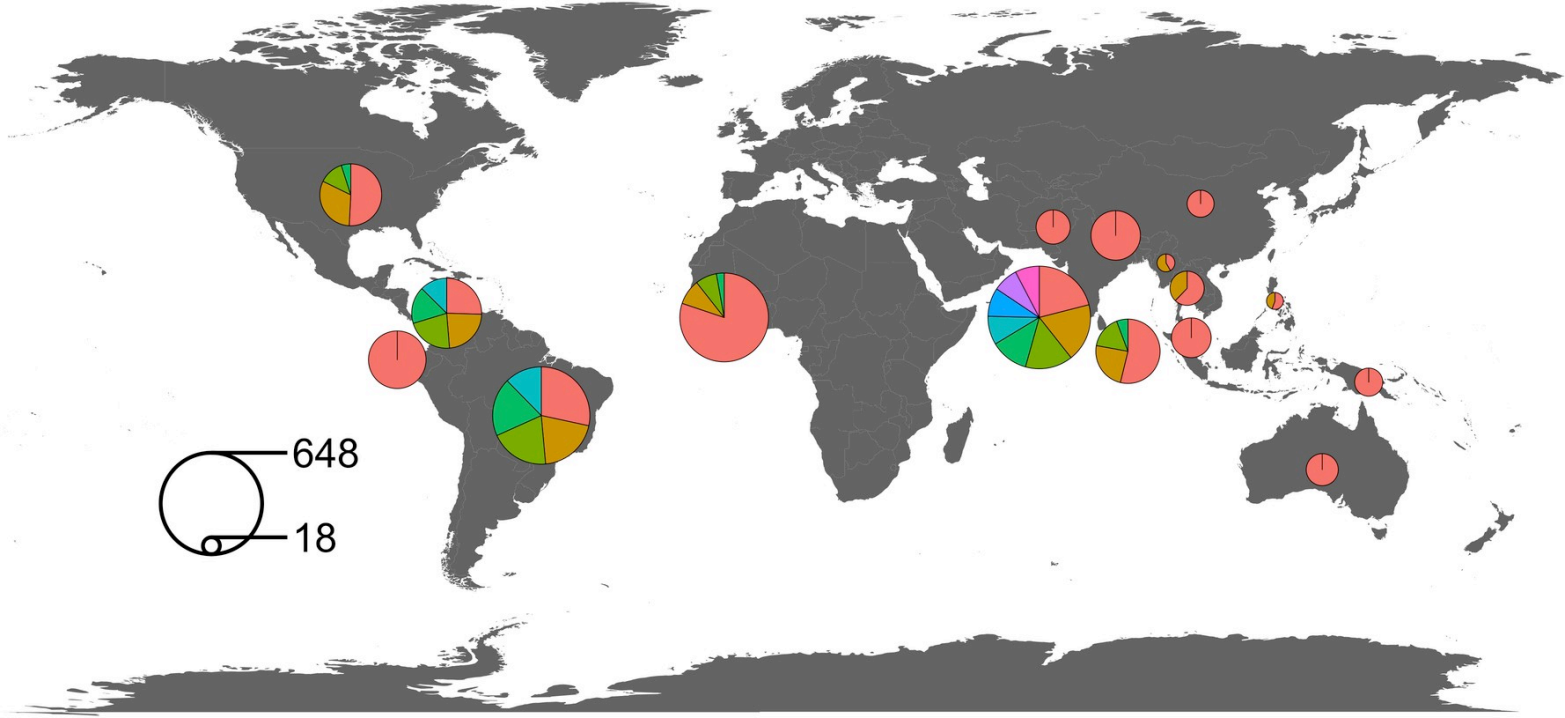
Total number of randomised controlled trial participants by country. The area of each circle is proportionate to the total number of trial participants randomised per country in studies published between 1946 and 2020. The area of the segments of each circle are proportionate to the sample size of individual RCTs (e.g., there has been one large trial and three small trials conducted in Nigeria). Each circle overlies the country that it refers to. Where circles would overlap they have been moved, and the edge of the circle touches the corresponding country. The key demonstrates the samples size that corresponds to the surface area of two example circles. World map sourced from the Natural Earth project (1:50m resolution version) https://www.naturalearthdata.com.

The proportion of randomised controlled trials conducted in each region were: Asia, 51·2% (n = 22 of 43); South America, 25·6% (n = 11); North America, 9·3% (n = 4); Africa, 9·3% (n = 4) (all in Nigeria); and Australasia, 4·7% (2) (Fig 2). The proportion published per decade were: 1960–69, 2·3% (n = 1 of 43); 1970–79, 2·3% (n = 1); 1980–89, 14·0% (n = 6); 1990–99, 27·9% (n = 12); 2000–09, 25·6% (n = 11); 2010–19, 25·6% (n = 11); and 2020, 2·3% (n = 1). Across the 43 trials, 3,418 participants were randomised. The mean sample size was 79 (IQR 41–100) and in 74·4% (n = 32 of 43) of studies the sample size was ≤100. The majority (55·8%; n = 24 of 43) were single centre studies, and the four trials with the greatest number of recruitment sites (range 7–28 sites) were conducted exclusively in Australia or the USA. Double blinding was adopted in 44·1% (n = 19 of 43); single blinding in 4·7% (n = 2); and 51·1% (n = 22) were open label. No pre-specified time-period of follow-up was defined in 55·8% (n = 24) trials. Among those where a follow-up period was reported (n = 19), 89.5% (n = 17) were for a period of 28 days or less; 5·3% (n = 1) were for 3-months; and 5·3% (n = 1) were for 6-months. Amongst trials published since January 2000 (N = 23), 43·5% (n = 10) reported a sample size calculation; 65·2% (n = 15) reported the numbers of participants screened for eligibility; and 43·5% (n = 10) were registered with a protocol available.

**Fig 3:**
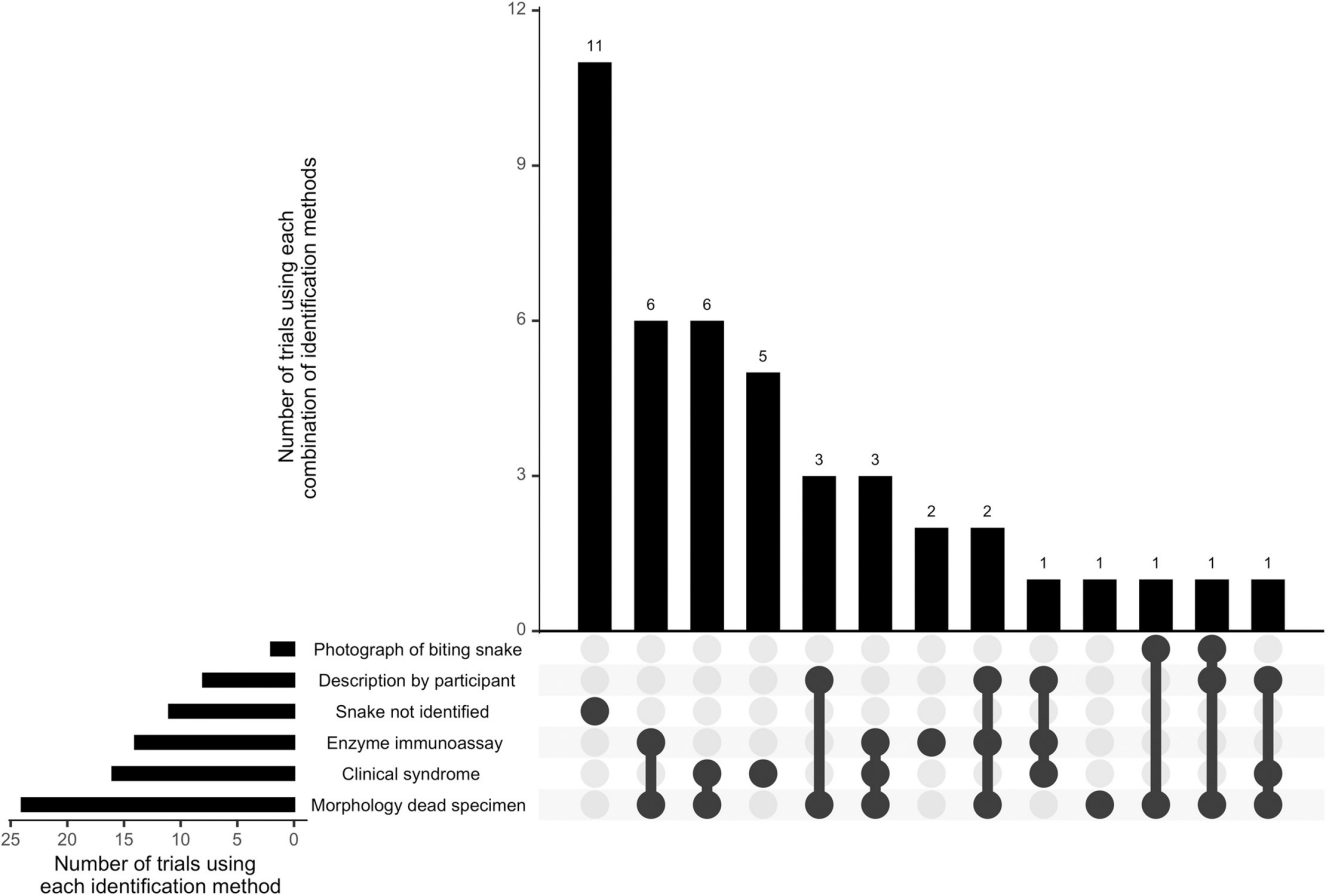
UpSet plot summarising methods of snake identification used in snakebite randomised controlled trials. Upper bar chart, x axis: combinations of snake identification methods; y axis: number of randomised controlled trials using each combination of snake identification methods. Lower left bar chart, x axis: total numbers of randomised controlled trials using each individual method of snake identification; y axis: individual methods of snake identification.

In 74·4% (n = 32 of 43) of randomised controlled trials, a method of identifying the biting snake, to species, genus or sub-family taxonomic rank, was used. The majority of trials combined two or more methods for identifying the snake. In 55·8% (n = 24) of clinical trials, the morphology of the dead snake was opportunistically assessed (when the specimen was brought into hospital), although other less specific methods of identification were often relied upon in these trials, such as an assessment of the clinical syndrome of envenoming. The clinical syndrome of envenoming (together with valid assumptions of locally prevalent snake species) was used to predict the biting species in 37·2% (n = 16) of clinical trials. Enzyme immunoassay, the participant’s description of the snake’s appearance, or a photograph of the biting snake (taken by the participant or a bystander) were assessed in 32·6% (n = 14), 18·6% (n = 8) and 4·7% (n = 2) of clinical trials, respectively. The UpSet plot (Fig 3) demonstrates the size of intersections between the different methods of snake identification used across the 43 included clinical trials. Amongst trials which identified the biting snake (n = 32), these were Viperidae in 87·5% (n = 28) of studies, and Elapidae in 12·5% (n = 4) of studies. The most commonly studied snake genera were *Bothrops* (34·4%; n = 11), *Daboia* (15·6%; n = 5) and *Echis* (12·1%; n = 4).

Participants with coagulopathy or haemorrhagic envenoming were studied in 81·4% of trials (n = 35 of 43); local tissue damage in 69·8% (n = 30); renal injury in 25·6% (n = 11); neurotoxicity in 20·9% (n = 9); and myotoxicity in 2·3% (n = 1). Different antivenom products were compared in 44·2% (n = 19) of trials; different doses of the same antivenom product were compared in 23·3% (n = 10) of trials. Antivenom was compared to placebo in 9·3% (n = 4) of trials; in all of these, participants with severe envenoming were excluded [15,16] or the biting species was known to be associated with limited clinical manifestations [17,18]. Other therapies that were compared were: heparin, 9·3% (n = 4) [19–22]; fresh frozen plasma, 4·7% (n = 2) [23,24]; atropine and edrophonium, 2·3% (n = 1) [25]; edrophonium and amifampridine, 2·3% (n = 1) [26]; intravenous immunoglobulin, 2·3% (n = 1) [27]; and ‘Qingwen Baidu Decoction’ (a traditional Chinese medicine), 2·3% (n = 1) [28].

### Quality of outcome measures

Amongst the trials and protocols (n = 56), 50·0% (n = 28) had a clearly stated primary outcome. Amongst studies published since January 2000 (n = 36), 69·4% (n = 25) had a clearly stated primary outcome. 80·0% (n = 20) of primary outcomes were clearly defined; 64% (n = 16) were clinical endpoints and 36% (n = 9) were laboratory markers; and 4·0% (n = 1) were patient centred. Across the secondary outcome measures from studies published since January 2000 (n = 226), 64·6% (n = 146) were clearly defined; 56.6% (n = 128) were clinical endpoints, 38.9% (n = 88) were laboratory markers, and 4.4% (n = 10) were exploratory; and 4·9% (n = 11) were patient centred.

### Outcome measures

**Fig 4:**
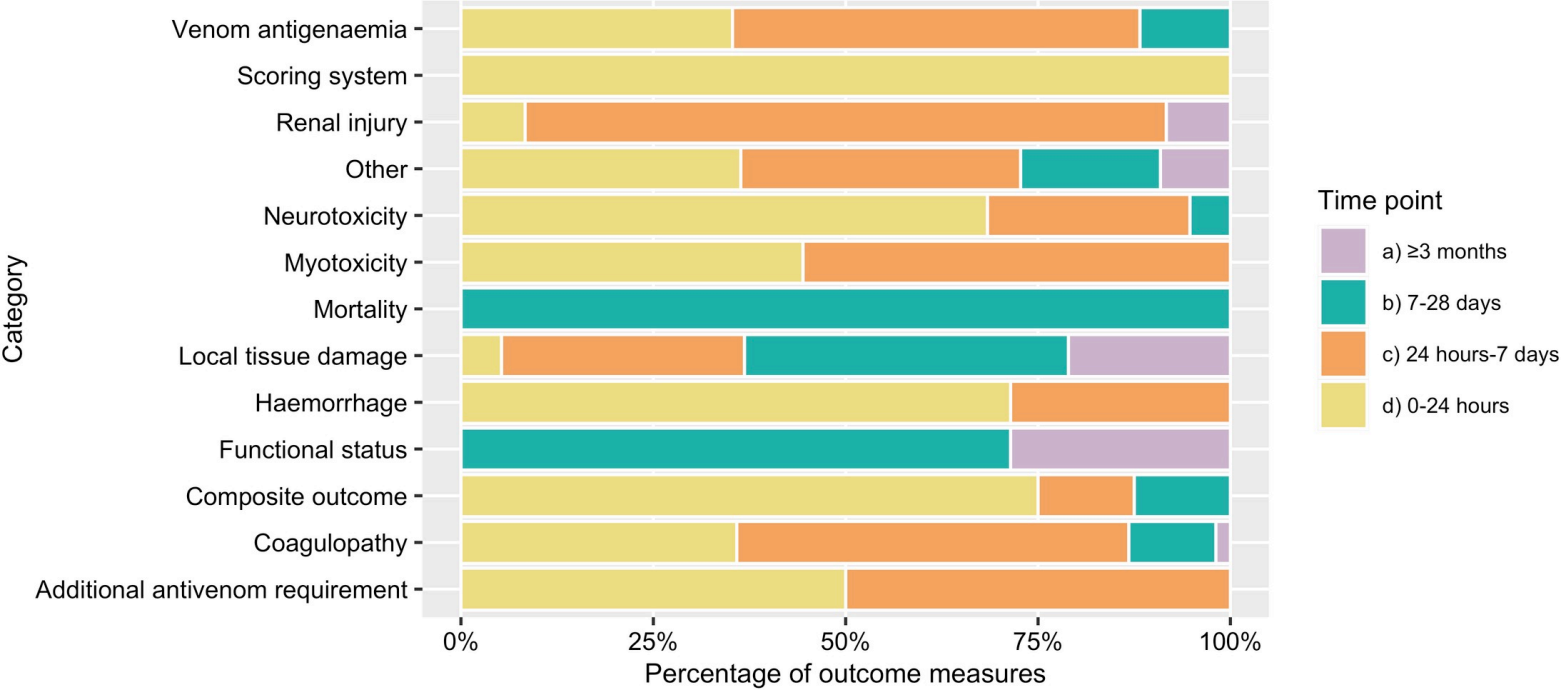
Duration of follow-up of outcome measures: grouped by category of envenoming. Fig 4 depicts the time-period of follow-up of outcome measures within each category. For each outcome measure, the latest time point of follow-up was identified. Time points were grouped as: up to 24 hours; up to 7 days; up to 28 days; up to 3 months; and over 3 months. Within each category, the proportion of outcome measures with follow-up until each time-point is defined. For example, mortality outcome measures were always followed up for over 7 days but were never followed up for more than 28 days. No outcome measures were followed up until between 28 days and 3 months, and therefore this time point is not displayed in Fig 4.

Across the 58 included studies, 382 outcome measures were extracted verbatim and, after duplicates were merged, 153 unique outcomes were identified. 59·5% of unique outcome measures were unique to a single study; 18·3% were used in two studies; 5·9%, in three studies; 4·6%, in four studies; and 11·8% were used in five or more studies. There was no single outcome that was used across all the studies, and the most frequently used outcome domain (‘venom antigenaemia’) was included in 32·8% of studies (n = 19 of 58). Venom antigenaemia was measured using various assays; the majority of which are not commercially available. 39·8% of the 382 extracted outcome measures did not report a specific timing of measurement. Amongst those with a time-point, 35·7% were measured for less than 24 hours; 42·6%, for less than a week; 16·5%, for less than a month; and 5·2% for up to 6-months. A summary of the durations of follow-up of outcome measures, grouped by category of envenoming, are presented in Fig 4. Full extracted outcome measure data is available in (S1 Data).

The 28 primary outcome measures were categorised as follows: composite outcome, 8; coagulopathy, 6; neurotoxicity, 4; local tissue damage, 2; additional antivenom requirement, 1; functional status, 1; myotoxicity, 1; renal injury, 1; scoring system, 1; venom antigenaemia, 1; and other, 2. Table 1 summarises these primary outcomes, and includes the verbatim data extraction. The outcomes categorised as ‘other’ included one of antivenom hypersensitivity reaction [29] and one which was poorly defined [30]. The majority of primary outcome measures (82·1%) were unique to a single study. Amongst studies with shared primary outcome measures, three by Isbister et al adopted return of INR to less than two [23,24,31] and two studies measured duration of invasive ventilation [32,33].

For the remainder of the analysis herein, the 153 unique primary and secondary outcome measures will be considered together. Outcome measures were classified into 60 domains, and these are detailed in their corresponding categories in Table 2.

**Table 2:** Overview of outcome categories and domains.

Category	Number of studies using an outcome measure within each category	Domain	Number of unique outcome measures within each domain	Number of studies using an outcome measure within each domain
Mortality	18	Mortality	1	18
Neurotoxicity	14	Ptosis	3	6
		Requirement for invasive ventilation	2	6
		Duration of invasive ventilation	3	4
		Extraocular muscle palsy	2	3
		Measure of other skeletal muscle weakness	2	3
		Bulbar palsy	3	2
		Electromyography	1	2
		Spirometry	3	1
		Neurotoxicity outcome poorly defined	1	2
Haemorrhage	18	Cessation of local or systemic bleeding	4	9
		Anaemia	3	4
		ISTH defined major bleeding	1	4
		Blood transfusion requirement	1	3
Coagulopathy	42	Bedside clotting test	18	30
		- 20-minute whole blood clotting test	(9)	(15)
		- Lee White clotting time	(6)	(12)
		- Bleeding time	(1)	(2)
		- Other bedside clotting assay	(2)	(3)
		Fibrinogen quantification	5	17
		Clotting studies (INR, PT, APTT)	9	14
		Platelet count	3	9
		Clotting factor quantification	6	7
		Fibrin and Fibrinogen-Degradation Products quantification	5	6
		Clotting factor replacement	1	2
Myotoxicity	7	Creatinine kinase	1	6
		Myoglobinuria	2	2
		Myalgia	1	1
Renal injury	14	Acute kidney injury (non-specific criteria)	4	6
		Requirement for renal replacement therapy	1	6
		Serum creatinine	2	5
		Serum urea	1	3
		Acute kidney injury (RIFLE or KDIGO criteria)	1	2
		Haematuria	1	1
		Renal outcome poorly defined	1	1
Local tissue damage	19	Development of skin blistering or necrosis	4	9
		Swelling measured by circumference of bitten limb	3	9
		Pain—ordinal scale	3	4
		Skin and soft tissue infection	4	4
		Opioid requirement	2	3
		Swelling measured as proximal extension	1	3
		Swelling measured by limb volume	1	2
		Need for amputation, skin grafting or debridement	3	1
		Local tissue damage outcome poorly defined	1	2
Cardiotoxicity	3	Hypotension	2	3
Venom antigenaemia	19	Venom antigen quantification	1	19
Additional antivenom requirement	10	Total dose of antivenom required	1	6
		Need for additional antivenom following initial dosing	3	4
Functional status	12	Measure of limb weakness	2	3
		Multipoint scale of physical function	8	3
		Number of therapy sessions	1	1
		Return to work	1	1
		Functional status outcome poorly defined	1	1
Scoring system	1	Snakebite severity score	1	1
Composite outcome	14	Composite outcome	13	13
Other	19	Duration of hospital admission	1	16
		Allergic reaction	1	1
		Anosmia	1	1
		GI symptoms of envenoming	2	1
		Hypoxic brain injury	1	1
		Leucocyte count	1	1
		Serum lactate dehydrogenase	1	1
		Serum metalloproteinase	1	1
		‘Other’ outcome poorly defined	1	7

Categories were predefined and each represent an established clinical syndrome of envenoming (e.g., neurotoxicity), or a broad categorisation of the outcome type (e.g., composite outcome). Two reviewers independently categorised outcome measures and consensus was reached on disagreements.

Domains were defined using a data driven approach. Each domain represents a grouping of outcomes that were deemed to be measuring a similar parameter. Consensus on domain allocations and domain names was reached by the primary authors.

APTT = activated partial thromboplastin time, GI = gastrointestinal, ISTH = International Society on Thrombosis and Haemostasis, INR = international normalised ratio, KDIGO = Kidney Disease: Improving Global Outcomes, and PT = prothrombin time.

Outcome measures in the category ‘coagulopathy’ were included in 72·4% of studies (n = 42 of 58). An outcome measure in the ‘bedside clotting test’ domain was used in 51·7% (n = 30) of studies and, within this domain, 18 unique methods of measurement were identified. These included various iterations of the ‘20-minute whole blood clotting test’, ‘Lee White clotting time’ and ‘bleeding time’, which were used in 15, 12 and 2 studies, respectively. The next most widely used coagulation domains were ‘fibrinogen quantification’ (29·3%; n = 17), and ‘clotting studies’ (24·1%; n = 14).

Outcome measures in the category ‘haemorrhage’ were adopted in 31·0% of studies (n = 18 of 58) and were grouped into the following domains: ‘cessation of local or systemic bleeding’; ‘anaemia’; ‘ISTH defined major bleeding’ and ‘blood transfusion requirement’. The most widely used was ‘cessation of local or systemic bleeding’ which was adopted in 15·5% of studies (n = 9) and was measured in four unique ways.

Neurotoxicity outcome measures were reported in 24·1% of studies. Amongst the 14 studies that used a neurotoxicity outcome measure, the most widely used were ‘ptosis’ (42·9% of studies); ‘requirement for invasive ventilation’ (42·9% of studies); and ‘duration of invasive ventilation’ (28·6% of studies). ‘Electromyography’ was used in 14·3% and ‘spirometry’ was used in 7·1% of studies with a neurotoxicity outcome measure.

Renal injury outcome measures were adopted in 24·1% of studies (n = 14 of 58) and were predominantly based on measurements of creatinine or urine output, with various cut-offs for defining abnormal. 5·2% (n = 3) of studies adopted the RIFLE or KDIGO criteria for defining acute kidney injury [34,35] including one study where it formed a component of a composite outcome [36]. The proportion of participants requiring renal replacement therapy were measured in 10·3% of studies (n = 6); all conducted in India [19,22,34,37–39].

Outcome measures that assessed local tissue damage were adopted in 32·8% of studies (n = 19 of 58). ‘Development of skin blistering or necrosis’ and ‘swelling measured by circumference of bitten limb’ were the most widely used, being included in 15.5% of studies (n = 9). The ‘need for amputation, skin grafting or debridement’ outcome domain was only adopted in one study.

The ‘multipoint scale of physical function’ domain was the most widely adopted measure of ‘functional status’ and included eight functional scales (as reported in S3 Text). All of these were patient-centred outcomes and were used in three studies; all conducted in the USA [13,15,40]. A scoring system, the ‘snakebite severity score’ [41], was used in a single study [42]. Composite outcomes were included in 13 studies, and each of these were unique and represented the primary outcome measures (Table 1).

### Adverse event outcome measures

Amongst the trials and protocols (n = 56), there was a failure to record adverse event outcomes in 32·1% (n = 18) of studies. A total of 69 adverse event outcome measures were extracted verbatim, and were grouped as follows: ‘anaphylaxis’, 18; ‘early hypersensitivity reactions’, 6; ‘non-specific early reactions, 19; ‘pyrogenic reactions’, 3; full adverse event reporting (reporting of all serious adverse events), 8; ‘transfusion-related acute lung injury’, 2; and ‘serum sickness’, 13. Anaphylaxis was defined based on published criteria in five trials or protocols [23,24,31,43,44]. Serum sickness was defined based on reproducible clinical criteria in one published randomised controlled trial, and one trial protocol [42,43].

## Discussion

Outcome measures used in clinical trials of snakebite envenoming vary considerably. Although varied outcome measures are needed to capture the diverse effects of envenoming by different species, variations within an outcome domain are undesirable. To achieve the WHO target of reducing snakebite deaths and disability by 50%, clinical trial outcome measures must include either direct measures of clinically relevant events or validated surrogate markers that are known to be associated with risk of disability or death.

This systematic review also demonstrates the troubling landscape of clinical trials in snakebite. Many recent trials did not use a sample size calculation, were single centre and were underpowered. Few clinical trials have been conducted in sub-Saharan Africa or the Middle East. Policy makers and clinicians are faced with a disturbing lack of data on which to evaluate antivenoms. Similar to our findings amongst randomised controlled trials of antivenoms, pre-clinical efficacy testing has used heterogenous methods that in a number of cases prevent comparisons between studies [45]. There is an urgent need for standardisation in the way that antivenoms are assessed, both pre-clinically and clinically.

Of further concern, many of the included clinical trials used unreliable methods for identifying the biting snake species. As the efficacy of antivenom is often snake species specific, knowing the biting species is important. Although the majority of trials utilised an assessment of the morphology of the dead specimen brought to the hospital, this was invariably opportunistic. For those participants who did not attend with the dead specimen, less specific methods were largely relied upon. Amongst eight of the 43 included clinical trials, participants were asked to recall and describe the appearance of the snake, and in a further 11 clinical trials no efforts were made to identify the biting species. The clinical syndrome of envenoming was used to predict the biting species in 16 clinical trials and, although this method can be reliable in settings where a single species is the predominant cause of coagulopathy, such as parts of West Africa, this is not reliable in various other settings. Unfortunately, reliable identification of the biting species remains challenging, particularly in LMIC settings, and further development of enzyme immunoassay and molecular based methods for snake identification are urgently needed.

There have been just nine randomised controlled trials that have included participants with neurotoxicity, with a combined sample size of 492 [25–27,32,38,39,46–48]. All except one study [26] took place in Asia. Many studies adopted measures of eyelid strength or requirement for mechanical ventilation. Outcomes used in other neuromuscular disorders may be useful. For example, the ‘myasthenic muscle score’ is a validated 100-point scale used to assess therapeutic efficacy in myasthenia gravis [49,50]. A scoring system has the advantage of capturing weakness of various muscle groups and providing a semi-quantitative measure that may more sensitively detect response to therapeutics. Spirometry, including measurement of forced vital capacity, offers a potentially sensitive and quantifiable measure of respiratory muscle strength and was used in one included study [25], although its validity in other neuromuscular disorders has been disappointing [51]. For phase III clinical trials, pragmatic endpoints with high clinical relevance will be important, such as the proportion of participants requiring intubation and ventilation.

Bleeding events were often poorly defined with insufficient detail to allow consistent replication in future studies [19,52–54]. Bleeding due to snake envenoming tends to involve small volume blood loss from the bite site, gums, or venepuncture sites. Although the International Society on Thrombosis and Haemostasis (ISTH) definition [55] of haemorrhage [23,24,31,36], or laboratory-based measures of anaemia [56–59], provide objective tools, bleeding events of this severity are rare in snakebite envenoming [60]. Furthermore, measures of haematocrit or packed cell volume [56–59] may under-estimate anaemia due to the concentration effect of venom-induced capillary leak syndrome [61].

A range of laboratory assays were used to assess for coagulopathy, and it is uncertain which is the most useful. The bedside clotting tests do not require any specialist equipment and can be conducted in remote rural settings where the majority of snakebites occur. The Lee White clotting time was used exclusively in studies from South America and Asia [19,37,38,62–67], whereas the 20-minute whole blood clotting test (20WBCT) has been used more widely [20,34,47,52–54,56,59,68–71]. Although the 20WBCT has been subject to more frequent validation than the Lee White clotting time, this has rarely been amongst participants that have received antivenom [24,72,73] and, therefore, bedside tests of whole blood clotting are inadequately validated for measuring response to treatment. A disadvantage of the 20WBCT is that it is binary rather than continuous. Sensitive continuous outcome measures are desirable for smaller studies such as phase II clinical trials.

Renal injury can result from envenoming by a range of snake species [74]. Outcome measures in snakebite clinical trials have focussed on acute renal injury; based on various thresholds of serum creatinine and oliguria. Internationally recognised criteria for the diagnosis of acute kidney injury are available [75–77], and these were adopted in three of the included studies [34,35], including within one composite outcome [36]. Although the need for renal replacement therapy (RRT) is an important measure, there is wide variation and no consensus on the optimal timing for initiating and stopping this in acute kidney injury [78]. Follow-up studies of adults and children with snake venom induced renal injury have demonstrated a 30% risk of progression to chronic kidney disease [79,80]. Chronic kidney disease is defined as an abnormality in the structure or functioning of the kidneys present for a minimum of 3 months [81]. No outcome measures fulfilled this definition and the need for longer follow-up of renal function in clinical trials should be considered.

Snakebite associated local tissue damage represents a varied spectrum of disease, ranging from swelling to necrosis, with complications including infection, contractures, and amputation. Although this range of disease was captured across the extracted outcomes, this was inconsistent between studies. Consensus on a list of outcomes, including details of how they should be measured, is needed. For example, limb swelling has been measured by circumference [18,27,35,47,63], distance of proximal extension [40,58,59], or limb volume [40,82].

Complications of local tissue damage can cause loss of physical function with a varying impact depending on an individual’s circumstances. Many people with snakebite are vulnerable and disability may significantly impact on their ability to work, subsistence farm or care for children. Patient-centred outcomes are key for capturing this, but such outcomes were only adopted in trials based in the USA [13,15,40]. The patient specific functional scale (PSFS) is simple (although does require numeracy) and allows patients to identify functions that are important to them. This tool has been validated for snakebite envenoming [13], although not in an LMIC setting.

Adverse event reporting varied significantly between the randomised controlled trials, and 32·1% of the included studies failed to report adverse events. As antivenom is an animal derived product, there is a significant risk of life-threatening anaphylaxis, yet only five of the 56 included studies used standardised published criteria for defining anaphylaxis. Given that the risk of anaphylaxis can vary substantially between antivenom products [7,83], it is essential that the rate of occurrence of these events can be reliably and consistently measured in clinical trials. Serum sickness was only reported as an outcome measure in 13 of the 58 included studies, and only two studies used clearly defined clinical criteria [42,43]. A standardised definition of anaphylaxis and serum sickness should be included in a core outcome set.

### Limitations

When considering outcome measures for use in a core outcome set, it is important to ascertain whether they are valid, reliable, and feasible. Such an assessment was outside the scope of this study and will form the next stage of COS development. This systematic review did not restrict on the age or quality of the trials; however, this was important to ensure all outcome measures were captured. When describing the characteristics and quality of trials, data for studies published more recently were presented. At this stage there has not been any patient involvement and future work on COS development will strive to involve people who have directly experienced snakebite.

## Conclusions

This study has identified significant heterogeneity of outcome measures in snakebite clinical trials. There is a strong need for a core outcome set, which will support the adoption of valid, reproducible, and patient-centred outcome measures, and enable downstream meta-analyses. Validated outcome measures are particularly important when assessing antivenom efficacy, as this expensive therapy is associated with a relatively high risk of adverse events. To provide global relevance that can span the diversity of snake species, outcomes that represent each of the syndromes of envenoming are needed. Through better outcome measures, together with increased global recognition of the importance of snakebite envenoming, high quality clinical trials in populations with the greatest burden of disease can be achieved.

## Supporting information

S1 Text Details of extracted data. (DOCX)

S2 Text Tool to assess methodological quality of outcome measures. (DOCX)

S3 Text Summary of all extracted multipoint scales of physical function. (DOCX)

S1 Data Full extracted outcome measure data. (CSV)
