# Supplementary material for: Clinical outcomes and outcome measurement tools reported in randomised controlled trials of treatment for snakebite envenoming: A systematic review
Source: PLoS Negl Trop Dis. 2021 Aug 2;15(8):e0009589. doi: 10.1371/journal.pntd.0009589 (PMC8360524; doi:10.1371/journal.pntd.0009589)
Supplement: S1 Text — (DOCX) [file pntd.0009589.s001.docx]

**S1 Details of extracted data**

The extracted data included: trial registration identification; title; first author; year published; year recruitment started and ended; country; number of sites; target sample size; number screened; number randomised; phase of trial (I-IV); blinding method; reported snake species and or genus; method of identifying snake; timeframe for inclusion; inclusion criteria; exclusion criteria; interventions group(s); control group(s); primary outcome measure (verbatim); secondary outcome measure (verbatim); time of assessment of outcome measures; quality assessment of outcome measures; adverse event outcome measures (verbatim); time of assessment of adverse event outcome measures; time between randomisation and the final follow-up visit. Where clinical trials did not clearly report a primary outcome, and where the trial protocol was available, the primary outcome reported in the protocol was recorded.
