# Supplementary material for: Clinical outcomes and outcome measurement tools reported in randomised controlled trials of treatment for snakebite envenoming: A systematic review
Source: PLoS Negl Trop Dis. 2021 Aug 2;15(8):e0009589. doi: 10.1371/journal.pntd.0009589 (PMC8360524; doi:10.1371/journal.pntd.0009589)
Supplement: S2 Text — (DOCX) [file pntd.0009589.s002.docx]

**S2 Tool to assess methodological quality of outcome measures**

The tool for assessing the quality of outcome measures and outcome reporting was adapted from the version published by Harman et al.(1)

1. Is a primary outcome clearly stated?
2. Is the primary outcome clearly defined so that another researcher would be able to reproduce it? (Where appropriate, this should include clear description of time points, the person measuring the outcome, how the outcome was measured (for example, tools and methods used) and where the outcome was measured)
3. Are the secondary outcomes clearly defined?
4. Do the outcomes have clinical significance? (reviewers selected one of the following)
5. Mortality OR life-threatening complication (such as requiring ventilation or major bleeding) OR complication associated with significant physical disability (such as limb amputation or cerebral bleed)
6. Psychological complication (such as a diagnosis of PTSD)
7. Minor clinical complication not reaching threshold for ‘a’ (limb swelling, minor bleeding [e.g., bleeding gums])
8. Laboratory parameter of clinical relevance (such as abnormal clotting or venom antigenaemia)
9. Exploratory parameter OR parameters of unproven clinical relevance (such as novel assays)
10. Is the outcome a patient oriented measure of health-related quality of life? (what the participant can do [such as activities of daily living or ability to work] or how they feel [data will usually be qualitative or based on a multipoint questionnaire]).

1. Harman NL, Bruce IA, Callery P, Tierney S, Sharif MO, O’Brien K, et al. MOMENT – Management of Otitis Media with Effusion in Cleft Palate: protocol for a systematic review of the literature and identification of a core outcome set using a Delphi survey. Trials. 2013 Mar 12;14(1):70.
