# Supplementary material for: Clinical outcomes and outcome measurement tools reported in randomised controlled trials of treatment for snakebite envenoming: A systematic review
Source: PLoS Negl Trop Dis. 2021 Aug 2;15(8):e0009589. doi: 10.1371/journal.pntd.0009589 (PMC8360524; doi:10.1371/journal.pntd.0009589)
Supplement: S3 Text — (DOCX) [file pntd.0009589.s003.docx]

**S3 Summary of all extracted multipoint scales of physical function**

1. American Academy of Orthopaedic Surgeons (AAOS) normative outcome score
2. American Medical Association (AMA) disability rating score
3. Disabilities of the arm, shoulder and hand score (DASH score)
4. lower extremity functional scale (LEMS)
5. Patient-reported outcome measurement information system physical function-10 score (PROMIS PF-10)
6. Patient-specific functional scale (PSFS)
7. Patient’s global impression of change-1 instrument
8. The physical function domain of the SF-36 questionnaire.
