# Supplementary material for: Clinical outcomes and outcome measurement tools reported in randomised controlled trials of treatment for snakebite envenoming: A systematic review
Source: PLoS Negl Trop Dis. 2021 Aug 2;15(8):e0009589. doi: 10.1371/journal.pntd.0009589 (PMC8360524; doi:10.1371/journal.pntd.0009589)
Supplement: Attachment — Submitted filename: Response letter to editor_v2.pdf [file pntd.0009589.s005.pdf]

LSTM
LIVERPOOL SCHOOL
OF TROPICAL MEDICINE

Pembroke Place,
Liverpool, L3 5QA, UK
Tel: +44(0)151 705 3100
Fax: +44(0)151 705 3370

www.lstmed.ac.uk

12st May 2021

Dear Dr Habib,

Thank you very much for overseeing the review of our manuscript ("Clinical outcomes and outcome measurement tools reported in randomised controlled trials of treatment for snakebite envenoming: a systematic review"), and many thanks to the reviewers for their helpful and considered comments.

Please find enclosed a revised manuscript (a version with tracked changes and a clean version) and see below our responses to the reviewers' comments.

Reviewer #1: 1. Why safety outcomes were not assessed?

Adverse event outcome measures were extracted during this systematic review. This is highlighted in the methods under the sub-heading 'data extraction'. The extracted adverse event outcome measures are listed in the results section under the sub-heading 'adverse event outcome measures.'

Reviewer #2: Appropriate methodology was used.

I could not find the review registered on PROSPERO - please check reference number

Suggest stating that review was registered prospectively

Apologies, we omitted the letters 'CRD' from the registration ID and this prevented the protocol from being identified when searching the database. Searching for CRD42020196160 does identify the protocol (https://www.crd.york.ac.uk/prospero/display_record.php?RecordID=196160). The identifier 'CRD42020196160' has now been included in the manuscript. The wording in the manuscript has been changed to highlight that the protocol was registered prospectively:

> Line 31: "The study was prospectively registered with PROSPERO: CRD42020196160."
>
> Line 119: "The protocol was prospectively registered with PROSPERO (CRD42020196160)."

Reviewer #3: The systematic review question was clearly stated. The definition of eligibility and exclusion criteria were clear, however the authors need to justify the inclusion of study on traditional Chinese medicine "reference 28". Is the active ingredient in traditional Chinese medicine known to have therapeutic effect that inhibit snake venom toxins?

We agree with the reviewer that the use of "Qingwen Baidu Decoction" for the treatment of snakebite envenoming is questionable and, as far as we are aware, this therapeutic is not supported by robust pre-clinical or clinical data. However, the assessment of the efficacy of potential therapeutics was outside the scope of this systematic review. As stated in the last sentence of the introduction, the aim was to formulate a comprehensive list of potential outcome measures:

> Line 73: "This systematic review aims to describe the heterogeneity in outcome measures used across clinical trials and will provide a comprehensive resource of outcome measures that can be considered when developing a COS."

The study assessing “Qingwen Baidu Decoction” did fulfil the inclusion and exclusion criteria. These criteria were developed prospectively and were listed in the protocol uploaded to PROSPERO. The outcome measures that were extracted from this study, and all included studies, underwent an independent assessment of quality, as outlined in Appendix B.

Reviewer #1: Chronic disabilities were also assessed? Any trial found? Important to discuss.

The trials reporting on outcome measures of disability are highlighted in the discussion section (line 342). Only a small number of clinical trials based in the USA measured outcome measures related to physical functioning.

> Line 340: *“Complications of local tissue damage can cause loss of physical function with a varying impact depending on an individual’s circumstances. Many people with snakebite are vulnerable and disability may significantly impact on their ability to work, subsistence farm or care for children. Patient centred outcomes are key for capturing this, but such outcomes were only adopted in trials based in the USA.[13,15,40]”*

These physical function scales are listed in Appendix D of the supplementary material:

> **Appendix D. Summary of all extracted multipoint scales of physical function**
>
> 1. American Academy of Orthopaedic Surgeons (AAOS) normative outcome score
> 2. American Medical Association (AMA) disability rating score
> 3. Disabilities of the arm, shoulder and hand score (DASH score)
> 4. lower extremity functional scale (LEMS)
> 5. Patient-reported outcome measurement information system physical function-10 score (PROMIS PF-10)
> 6. Patient-specific functional scale (PSFS)
> 7. Patient’s global impression of change-1 instrument
> 8. The physical function domain of the SF-36 questionnaire.

Reviewer #2: Consider combining Tables 1&2. If possible, i think also assessment of toxicity should be added to the trial.

As suggested by the reviewer, Table 1 has been adapted to include the following additional information from table 2: primary outcome measure (verbatim); whether the primary outcome was clearly defined; and whether the primary outcome was patient-centred. Furthermore, we have added an additional column listing the adverse event outcome domains that were included in each study. Table 2 (titled: “Primary outcome measures”) has been removed.

In updating the adverse event outcome data for Table 1, we adapted the grouping of domains to include ‘non-specific early reactions’ and ‘early hypersensitivity reactions’, instead of the previously combined item ‘early reactions.’ Five studies that were previously categorised as not reporting an adverse event outcome measure have been reclassified as reporting ‘non-specific early reactions.’ Furthermore, an error in the data was identified and one further study has been reclassified as recording TRALI as an adverse event outcome measure.

We have also added a statement highlighting that serum sickness was rarely defined using reproducible clinical criteria (only two studies).

The above changes are found in the last paragraph of the results section:

Line 256: Amongst the trials and protocols (n=56), there was a failure to record adverse event outcomes in 32·1% (n=18) of studies. A total of 69 adverse event outcome measures were extracted verbatim, and were grouped as follows: 'anaphylaxis', 18; 'early hypersensitivity reactions', 6; 'non-specific early reactions, 19; 'pyrogenic reactions', 3; full adverse event reporting (reporting of all serious adverse events), 8; 'transfusion-related acute lung injury', 2; and 'serum sickness', 13. Anaphylaxis was defined based on published criteria in five trials or protocols.[23,24,31,43,44] Serum sickness was defined based on reproducible clinical criteria in one published randomised controlled trial, and one trial protocol.[42,43]

Rows in Table 1 are numbered but not numbered in Table 2.

These numbers were retained in error and have now been removed from Table 1.

Data in text is expressed as %. I would prefer to see the absolute number and denominator.

We have now included the absolute number of studies in brackets adjacent to each percentage. The corresponding denominator is presented once at the beginning of each paragraph.

Abbreviation 20MWBCT is normally expressed as 20WBCT (see WHO SEARO Management of snakebite guidelines). 20WBCT - is abbreviation of 20-minute whole blood clotting test. As you say describe in the text, this is a binary outcome (clotted/unclotted) and not a time.

We thank the reviewer for identifying this error that we have now corrected throughout the manuscript.

Reviewer #3: The analysis presented was appropriate. There is need for authors to comment on the outcome measures used by studies with highest quality (these are randomize, double blinded and adequately powered studies).

The purpose of our study was to collate the outcome measures used in randomised controlled trials, of treatments for human snakebite, to provide a comprehensive resource for researchers, and to be the foundation of core outcome set development that would be relevant to high and low-income settings. This approach enabled a broad geographic focus and maximised the variety of snake species. Presenting an analysis of outcome measures for a subgroup of higher quality (from one perspective) studies, we do not feel would add to the value of our findings as this may lead the reader to infer that the outcome measures are also of higher quality, which is not necessarily the case, and it is likely that this would also result in restricted geographic focus and snake species inclusion. Assessing the quality of snakebite clinical trials is therefore outside the scope of this study, as is highlighted in the aim:

Line 73: "This systematic review aims to describe the heterogeneity in outcome measures used across clinical trials and will provide a comprehensive resource of outcome measures that can be considered when developing a COS."

Rather than assess the quality of each clinical trial, we used the tool in Appendix B to assess the quality of every extracted outcome measure.

Reviewer #1: Would it be possible today to carry out meta-analyses on the efficacy of the treatment of snakebites?

The aim of the study was to describe the heterogeneity in outcome measures used across clinical trials rather than determine the efficacy of treatments. However, although outside the scope of our systematic review, our impression is that it would not be possible to conduct a meta-analysis. This is supported by the Cochrane review published by Maduwage et al in 2015, which identified no studies fulfilling their inclusion criteria. This

is in part due to the heterogeneity in outcome measure choice, but also due to the variety of antivenom products and biting species that have been assessed, and the overall low number of clinical trials conducted.

Reviewer #1: For which species of snakes do we have better evidence in terms of the outcomes chosen in the trials?

We have reported on the number of trials recruiting participants with bites by each genera of snake:

> Line 169: "The most commonly studied snake genera were *Bothrops* (34·4%; n=11), *Daboia* (15·6%; n=5) and *Echis* (12·1%; n=4)."

Hence the trial outcome measures for those snake species have contributed more to our overall outcome measures, although we are not able to comment if there is better evidence for the use of certain outcome measures in particular species.

Reviewer #2: I think it is important to highlight the value of internationally accepted criterion for assessing preclinical efficacy of antivenom e.g. ED50 (Annex 5, WHO Guidelines for the production, control and regulation of snake antivenom immunoglobulins, p315) and how this has enabled comparitive efficacy testing across studies.

We agree that standardised methods for the pre-clinical efficacy testing of antivenoms is important. If this is acceptable to the reviewer, we have highlighted that the landscape of pre-clinical efficacy testing is similar to clinical trials: methods lack standardisation. We have referenced a recent systematic review of pre-clinical efficacy data for African antivenoms:
https://journals.plos.org/plosntds/article?id=10.1371/journal.pntd.0008579

> Line 274: Policy makers and clinicians are faced with a disturbing lack of data on which to evaluate antivenoms. Similar to our findings amongst randomised controlled trials of antivenoms, pre-clinical efficacy testing has used heterogenous methods that in a number of cases prevent comparisons between studies.[45] There is an urgent need for standardisation in the way that antivenoms are assessed, both pre-clinically and clinically.

Reviewer #2: The reporting of adverse reactions to antivenom is alarming and warrants discussion. I believe that a COS for snakebite trials must include a prospective definition of toxicity.

To better highlight the poor reporting of adverse events in snakebite clinical trials, we have added the following paragraph to the discussion section:

> Line 347: Adverse event reporting varied significantly between the randomised controlled trials, and 32·1% of the included studies failed to report adverse events. As antivenom is an animal derived product, there is a significant risk of life-threatening anaphylaxis, yet only five of the 56 included studies used standardised published criteria for defining anaphylaxis. Given that the risk of anaphylaxis can vary substantially between antivenom products,[7,83] it is essential that the rate of occurrence of these events can be reliably and consistently measured in clinical trials. Serum sickness was only reported as an outcome measure in 13 of the 58 included studies, and only two studies used clearly defined clinical criteria.[42,43] A standardised definition of anaphylaxis and serum sickness should be included in a core outcome set.

Reviewer #2: Albeit beyond the scope of this review, i think it worth discussing the importance of accurately identifying the responsible snake in snakebite trials (e.g. EIA or dead snakes). Reliance on syndrome and patient history is not sufficiently accurate for an RCT.

In response to the reviewer's suggestion to provide more detail on the assessment of the biting species, we have added an UpSet plot (Figure 3). This method of depicting the data was chosen as the majority of the clinical trials utilised two or more methods of identifying the biting species. Many trials assessed morphology of the dead specimen opportunistically (when participants attended with the dead specimen) and used a less reliable method for the majority of cases who did not attend with the dead specimen. The UpSet plot demonstrates the overlapping use of more and less reliable methods of snake identification.

Figure 3. UpSet plot summarising methods of snake identification used in snakebite randomised controlled trials

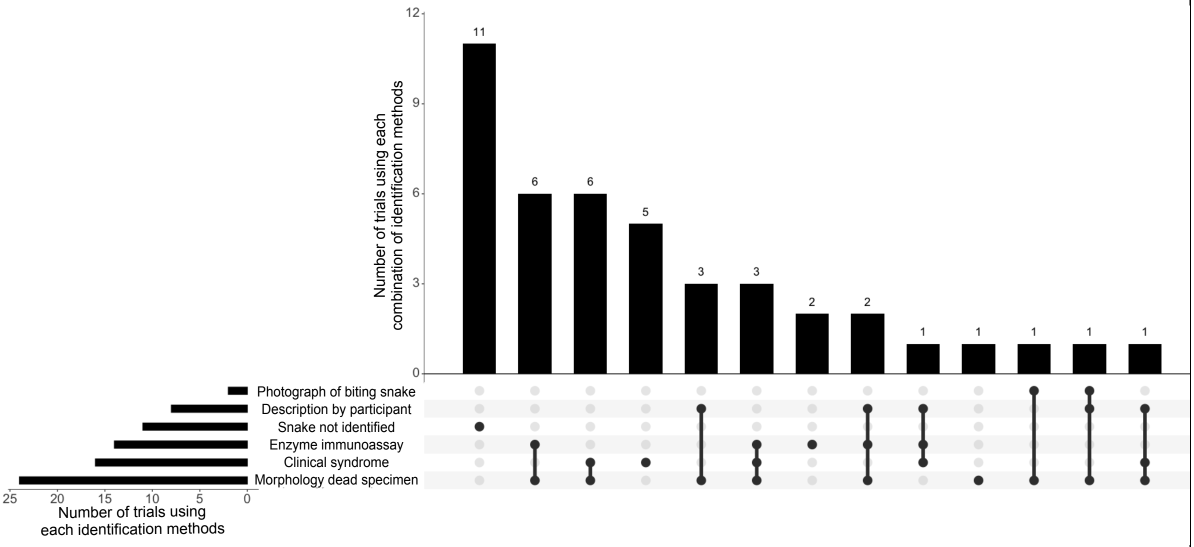

Upper bar chart, x axis: combinations of snake identification methods; y axis: number of randomised controlled trials using each combination of snake identification methods.
Lower left bar chart, x axis: total numbers of randomised controlled trials using each individual method of snake identification; y axis: individual methods of snake identification.

The following text has been added to the results section:

Line 158: In 74·4% (n=32 of 43) of randomised controlled trials, a method of identifying the biting snake, to species, genus or sub-family taxonomic rank, was used. The majority of trials combined two or more methods for identifying the snake. In 55·8% (n=24) of clinical trials, the morphology of the dead snake was opportunistically assessed (when the specimen was brought into hospital), although other less specific methods of identification were often relied upon in these trials, such as an assessment of the clinical syndrome of envenoming. The clinical syndrome of envenoming (together with valid assumptions of locally prevalent snake species) was used to predict the biting species in 37·2% (n=16) of clinical trials. Enzyme immunoassay, the participant's description of the snake's appearance, or a photograph of the biting snake (taken by the participant or a bystander) were assessed in 32·6% (n=14), 18·6% (n=8) and 4·7% (n=2) of clinical trials, respectively. The UpSet plot (Figure 3) demonstrates the size of intersections between the different methods of snake identification used across the 43 included clinical trials.

We have added the following paragraph to the discussion, highlighting the importance and challenges of snake species identification in clinical trials:

Line 280: Of further concern, many of the included clinical trials used unreliable methods for identifying the biting snake species. As the efficacy of antivenom is often snake species specific, knowing the biting species is important. Although the majority of trials utilised an assessment of the morphology of the dead specimen brought to the hospital, this was invariably opportunistic. For those participants who did not attend with

the dead specimen, less specific methods were largely relied upon. Amongst eight of the 43 included clinical trials, participants were asked to recall and describe the appearance of the snake, and in a further 11 clinical trials no efforts were made to identify the biting species. The clinical syndrome of envenoming was used to predict the biting species in 16 clinical trials and, although this method can be reliable in settings where a single species is the predominant cause of coagulopathy, such as parts of West Africa, this is not reliable in various other settings. Unfortunately, reliable identification of the biting species remains challenging, particularly in LMIC settings, and further development of enzyme immunoassay and molecular based methods for snake identification are urgently needed.

Reviewer #2: Line 280-282 discussed the prior validation of the 20WBCT. It should be highlighted that validation has occurred predominantly in antivenom naive patients. The 20WBCT or Lee White clotting method has not been sufficiently validated for assessing response to therapy.

We agree with the reviewer's comment and this statement has been modified to highlight that bedside whole blood clotting tests have not been validated as measures of response to treatment.

Line 317: Although the 20WBCT has been subject to more frequent validation than the Lee White clotting time, this has rarely been amongst participants that have received antivenom[24,72,73] and, therefore, bedside tests of whole blood clotting are inadequately validated for measuring response to treatment.

Reviewer #2: I disagree that a continuous outcome assessment is required to assess antivenom efficacy or toxicity (line 282-283).

We agree with the reviewer's comment and have changed the wording to highlight that continuous outcome measures are desirable for smaller phase II clinical trials.

Line 320: Sensitive continuous outcome measures are desirable for smaller studies such as phase II clinical trials.

Reviewer #2: I think it is worth noting early in the discussion what an ideal outcome measure should like - and how this may vary dependant on snake and level of healthcare. Most snakebite mortality and morbidity occurs in LMIC a COD needs to reflect this with a focus on clinical relevance. This is a clear benefit of using assessment such as 'need for ventillation' or 'need for RRT' or 'need for repeated doses of antivenom', providing the indication for such interventions are specified a priori.

The following statement has been added to the first paragraph of the discussion to highlight the importance of using outcome measures of clinical importance, or validated surrogate markers:

Line 268: To achieve the WHO target of reducing snakebite deaths and disability by 50%, clinical trial outcome measures must include either direct measures of clinically relevant events or validated surrogate markers that are known to be associated with risk of disability or death.

Reviewer #2: The citations in text do not match up with the references. For example the concentration effect of CLS (line 274) – is not supported by reference 60. Please check all citations and references.

Apologies for this oversight: we have refreshed the references, and these are now correctly linked.

The statement in line "72-73" seems incomplete.

This sentence has been better defined in response to the reviewer's comment, as follows:

> Line 71: By using a core outcome set, it is easier to compare, contrast and combine results of clinical trials, which has rarely been possible in the field of snakebite.

Reviewer #1: Present the common outcomes found in the articles included, in the Abstract.

The most frequently occurring outcome domain ('venom antigenaemia') has been added to the abstract:

> Line 38: The most frequently used outcome domain ('venom antigenaemia') was included in less than one third of the studies.

Reviewer #1: Is it possible to present a box with recommendations for future clinical trials, based on the findings of this study?

A subsequent project aims to develop a core outcome set for use in future clinical trials (https://www.comet-initiative.org/Studies/Details/1850). A robust approach to reaching consensus on a core outcome set is being used, and we have therefore limited our recommendations for future clinical trials in the discussion section of the present systematic review.

Reviewer #2: The authors should be commended for this valuable piece of work which has highlighted the need for COS in snakebite.

We thank the reviewer for their kind words.

Reviewer #3: The systematic review is timely, the findings shows significant heterogeneity in outcome measures in snakebite clinical trials. This justify development of globally relevant core outcomes set for snakebite clinical trials. However, the major weakness of the review is failure to assess the quality of the trials.

We thank the reviewer for the comment. We have explained about the reasoning for this in more detail above. Our systematic review omitted an assessment of clinical trial quality as we felt this was not relevant to our stated aim which was focussed on building a comprehensive list of outcome measures to inform a core outcome set. Rather than assess the quality of each clinical trial, we used the tool in Appendix B to assess the quality of every extracted outcome measure. This approach has been used in previous similar systematic reviews in other disease areas (https://bmjopen.bmj.com/content/9/2/e025135; doi: 10.1136/bmjopen-2018-025135).

Sincerely,

Dr Michael Abouyannis MRCP
Centre for Snakebite Research and Interventions
