# Supplementary material for: Clinical outcomes and outcome measurement tools reported in randomised controlled trials of treatment for snakebite envenoming: A systematic review
Source: PLoS Negl Trop Dis. 2021 Aug 2;15(8):e0009589. doi: 10.1371/journal.pntd.0009589 (PMC8360524; doi:10.1371/journal.pntd.0009589)
Supplement: Peer review history

# Peer review: Decision Letter 0

**DOI:** 10.1371/journal.pntd.0009589.r001

**Authors:** Abdulrazaq G. Habib, Kempaiah Kemparaju

22 Apr 2021

Dear Dr Esmail,

Thank you very much for submitting your manuscript "Clinical outcomes and outcome measurement tools reported in randomised controlled trials of treatment for snakebite envenoming: a systematic review" for consideration at PLOS Neglected Tropical Diseases. As with all papers reviewed by the journal, your manuscript was reviewed by members of the editorial board and by several independent reviewers. In light of the reviews (below this email), we would like to invite the resubmission of a significantly-revised version that takes into account the reviewers' comments. 

We cannot make any decision about publication until we have seen the revised manuscript and your response to the reviewers' comments. Your revised manuscript is also likely to be sent to reviewers for further evaluation.

When you are ready to resubmit, please upload the following:

[1] A letter containing a detailed list of your responses to the review comments and a description of the changes you have made in the manuscript. Please note while forming your response, if your article is accepted, you may have the opportunity to make the peer review history publicly available. The record will include editor decision letters (with reviews) and your responses to reviewer comments. If eligible, we will contact you to opt in or out.

[2] Two versions of the revised manuscript: one with either highlights or tracked changes denoting where the text has been changed; the other a clean version (uploaded as the manuscript file). 

Important additional instructions are given below your reviewer comments.

Please prepare and submit your revised manuscript within 60 days. If you anticipate any delay, please let us know the expected resubmission date by replying to this email. Please note that revised manuscripts received after the 60-day due date may require evaluation and peer review similar to newly submitted manuscripts.

Thank you again for your submission. We hope that our editorial process has been constructive so far, and we welcome your feedback at any time. Please don't hesitate to contact us if you have any questions or comments.

Sincerely,

Abdulrazaq G. Habib

Guest Editor

PLOS Neglected Tropical Diseases

Kempaiah Kemparaju

Deputy Editor

PLOS Neglected Tropical Diseases

***********************

Reviewer's Responses to Questions

**Key Review Criteria Required for Acceptance?**

As you describe the new analyses required for acceptance, please consider the following:

**Methods**

-Are the objectives of the study clearly articulated with a clear testable hypothesis stated?

-Is the study design appropriate to address the stated objectives?

-Is the population clearly described and appropriate for the hypothesis being tested?

-Is the sample size sufficient to ensure adequate power to address the hypothesis being tested?

-Were correct statistical analysis used to support conclusions?

-Are there concerns about ethical or regulatory requirements being met?

Reviewer #1: 1. Why safety outcomes were not assessed?

Reviewer #2: Appropriate methodology was used.

I could not find the review registered on PROSPERO - please check reference number

Suggest stating that review was registered prospectively

Reviewer #3: The systematic review question was clearly stated. The definition of eligibility and exclusion criteria were clear, however the authors need to justify the inclusion of study on traditional Chinese medicine "reference 28". Is the active ingredient in traditional Chinese medicine known to have therapeutic effect that inhibit snake venom toxins?

--------------------

**Results**

-Does the analysis presented match the analysis plan?

-Are the results clearly and completely presented?

-Are the figures (Tables, Images) of sufficient quality for clarity?

Reviewer #1: 1. Chronic disabilities were also assessed? Any trial found? Important to discuss.

Reviewer #2: Consider combining Tables 1&2. If possible, i think also assessment of toxicity should be added to the trial.

Rows in Table 1 are numbered but not numbered in Table 2.

Data in text is expressed as %. I would prefer to see the absolute number and denominator

Abbreviation 20MWBCT is normally expressed as 20WBCT (see WHO SEARO Management of snakebite guidelines).

20WBCT - is abbreviation of 20-minute whole blood clotting test. As you say describe in the text, this is a binary outcome (clotted/unclotted) and not a time.

Reviewer #3: The analysis presented was appropriate. There is need for authors to comment on the outcome measures used by studies with highest quality (these are randomize, double blinded and adequately powered studies).

--------------------

**Conclusions**

-Are the conclusions supported by the data presented?

-Are the limitations of analysis clearly described?

-Do the authors discuss how these data can be helpful to advance our understanding of the topic under study?

-Is public health relevance addressed?

Reviewer #1: 1. Would it be possible today to carry out meta-analyses on the efficacy of the treatment of snakebites?

2. For which species of snakes do we have better evidence in terms of the outcomes chosen in the trials?

Reviewer #2: I think it is important to highlight the value of internationally accepted criterion for assessing preclinical efficacy of antivenom e.g. ED50 (Annex 5, WHO Guidelines for the production, control and regulation of snake antivenom immunoglobulins, p315) and how this has enabled comparitive efficacy testing across studies. 

The reporting of adverse reactions to antivenom is alarming and warrants discussion. I believe that a COS for snakebite trials must include a prospective definition of toxicity.

Albeit beyond the scope of this review, i think it worth discussing the importance of accurately identifying the responsible snake in snakebite trials (e.g. EIA or dead snakes). Reliance on syndrome and patient history is not sufficiently accurate for an RCT.

Line 280-282 discussed the prior validation of the 20WBCT. It should be highlighted that validation has occurred predominantly in antivenom naive patients. The 20WBCT or Lee White clotting method has not been sufficiently validated for assessing response to therapy. 

I disagree that a continuous outcome assessment is required to assess antivenom efficacy or toxicity (line 282-283).

I think it is worth noting early in the discussion what an ideal outcome measure should like - and how this may vary dependant on snake and level of healthcare. Most snakebite mortality and morbidity occurs in LMIC a COD needs to reflect this with a focus on clinical relevance. This is a clear benefit of using assessment such as 'need for ventillation' or 'need for RRT' or 'need for repeated doses of antivenom', providing the indication for such interventions are specified a priori.

Reviewer #3: Conclusion supported by the data presented and limitation of the review was clearly stated.

--------------------

**Editorial and Data Presentation Modifications?**

Use this section for editorial suggestions as well as relatively minor modifications of existing data that would enhance clarity. If the only modifications needed are minor and/or editorial, you may wish to recommend “Minor Revision” or “Accept”. 

Reviewer #1: no

Reviewer #2: The citations in text do not match up with the references. For example the concentration effect of CLS (line 274) – is not supported by reference 60. Please check all citations and references.

Reviewer #3: The review requires minor revision.

The statement in line "72-73" seems incomplete.

--------------------

**Summary and General Comments**

Use this section to provide overall comments, discuss strengths/weaknesses of the study, novelty, significance, general execution and scholarship. You may also include additional comments for the author, including concerns about dual publication, research ethics, or publication ethics. If requesting major revision, please articulate the new experiments that are needed.

Reviewer #1: 1. Present the common outcomes found in the articles included, in the Abstract.

2. Is it possible to present a box with recommendations for future clinical trials, based on the findings of this study?

Reviewer #2: The authors should be commended for this valuable piece of work which has highlighted the need for COS in snakebite.

Reviewer #3: The systematic review is timely, the findings shows significant heterogeneity in outcome measures in snakebite clinical trials. This justify development of globally relevant core outcomes set for snakebite clinical trials.

However, the major weakness of the review is failure to assess the quality of the trials.

--------------------

PLOS authors have the option to publish the peer review history of their article (what does this mean?). If published, this will include your full peer review and any attached files.

If you choose “no”, your identity will remain anonymous but your review may still be made public.

**Do you want your identity to be public for this peer review?** For information about this choice, including consent withdrawal, please see our Privacy Policy.

Reviewer #1: No

Reviewer #2: Yes: Thomas Lamb

Reviewer #3: No

Figure Files:

While revising your submission, please upload your figure files to the Preflight Analysis and Conversion Engine (PACE) digital diagnostic tool, https://pacev2.apexcovantage.com. PACE helps ensure that figures meet PLOS requirements. To use PACE, you must first register as a user. Then, login and navigate to the UPLOAD tab, where you will find detailed instructions on how to use the tool. If you encounter any issues or have any questions when using PACE, please email us at figures@plos.org.

Data Requirements:

Please note that, as a condition of publication, PLOS' data policy requires that you make available all data used to draw the conclusions outlined in your manuscript. Data must be deposited in an appropriate repository, included within the body of the manuscript, or uploaded as supporting information. This includes all numerical values that were used to generate graphs, histograms etc.. For an example see here: http://www.plosbiology.org/article/info%3Adoi%2F10.1371%2Fjournal.pbio.1001908#s5.

Reproducibility:

To enhance the reproducibility of your results, we recommend that you deposit your laboratory protocols in protocols.io, where a protocol can be assigned its own identifier (DOI) such that it can be cited independently in the future. Additionally, PLOS ONE offers an option to publish peer-reviewed clinical study protocols. Read more information on sharing protocols at https://plos.org/protocols?utm_medium=editorial-email&utm_source=authorletters&utm_campaign=protocols

# Author comment: Author response to Decision Letter 0

**DOI:** 10.1371/journal.pntd.0009589.r002

28 May 2021

Attachment Submitted filename: Response letter to editor_v2.pdf Click here for additional data file.

# Peer review: Decision Letter 1

**DOI:** 10.1371/journal.pntd.0009589.r003

**Authors:** Abdulrazaq G. Habib, Kempaiah Kemparaju

24 Jun 2021

Dear Dr. Abouyannis,

We are pleased to inform you that your manuscript 'Clinical outcomes and outcome measurement tools reported in randomised controlled trials of treatment for snakebite envenoming: a systematic review' has been provisionally accepted for publication in PLOS Neglected Tropical Diseases.

Before your manuscript can be formally accepted you will need to complete some formatting changes, which you will receive in a follow up email. A member of our team will be in touch with a set of requests.

Please note that your manuscript will not be scheduled for publication until you have made the required changes, so a swift response is appreciated.

IMPORTANT: The editorial review process is now complete. PLOS will only permit corrections to spelling, formatting or significant scientific errors from this point onwards. Requests for major changes, or any which affect the scientific understanding of your work, will cause delays to the publication date of your manuscript.

Should you, your institution's press office or the journal office choose to press release your paper, you will automatically be opted out of early publication. We ask that you notify us now if you or your institution is planning to press release the article. All press must be co-ordinated with PLOS.

Thank you again for supporting Open Access publishing; we are looking forward to publishing your work in PLOS Neglected Tropical Diseases.

Best regards,

Abdulrazaq G. Habib

Guest Editor

PLOS Neglected Tropical Diseases

Kempaiah Kemparaju

Deputy Editor

PLOS Neglected Tropical Diseases

***********************************************************

Reviewer's Responses to Questions

**Key Review Criteria Required for Acceptance?**

As you describe the new analyses required for acceptance, please consider the following:

**Methods**

-Are the objectives of the study clearly articulated with a clear testable hypothesis stated?

-Is the study design appropriate to address the stated objectives?

-Is the population clearly described and appropriate for the hypothesis being tested?

-Is the sample size sufficient to ensure adequate power to address the hypothesis being tested?

-Were correct statistical analysis used to support conclusions?

-Are there concerns about ethical or regulatory requirements being met?

Reviewer #1: The authors adequately answered all my questions. I suggest that the manuscript can be accepted.

Reviewer #2: (No Response)

**********

**Results**

-Does the analysis presented match the analysis plan?

-Are the results clearly and completely presented?

-Are the figures (Tables, Images) of sufficient quality for clarity?

Reviewer #1: The authors adequately answered all my questions. I suggest that the manuscript can be accepted.

Reviewer #2: Thank you for incorporating the suggested changes. I believe the changes make the information easier to interpret and in the case of Table 1, more informative.

**********

**Conclusions**

-Are the conclusions supported by the data presented?

-Are the limitations of analysis clearly described?

-Do the authors discuss how these data can be helpful to advance our understanding of the topic under study?

-Is public health relevance addressed?

Reviewer #1: The authors adequately answered all my questions. I suggest that the manuscript can be accepted.

Reviewer #2: Thank you for considering my comment on comparing clinical outcomes to pre-clinical outcomes. I think there may have been some misunderstanding in my earlier comment.

Contrary to the statement written in the discussion, paragraph 2

"Similar to our findings amongst randomised controlled trials of antivenoms, pre-clinical efficacy testing has used heterogenous methods that in a number of cases prevent comparisons between studies."

I believe there is greater homogeneity in pre-clinical studies (e.g. use of ED50), which may in part be related to a published WHO framework for assessing clinical testing (Annex 5, WHO Technical Report Series). The authors may want to state how a consensus framework for the conduct of clinical antivenom studies (similar to that included in the the WHO Technical Report Series) may improve clinical outcome reporting in clinical studies.

**********

**Editorial and Data Presentation Modifications?**

Use this section for editorial suggestions as well as relatively minor modifications of existing data that would enhance clarity. If the only modifications needed are minor and/or editorial, you may wish to recommend “Minor Revision” or “Accept”.

Reviewer #1: The authors adequately answered all my questions. I suggest that the manuscript can be accepted.

Reviewer #2: (No Response)

**********

**Summary and General Comments**

Use this section to provide overall comments, discuss strengths/weaknesses of the study, novelty, significance, general execution and scholarship. You may also include additional comments for the author, including concerns about dual publication, research ethics, or publication ethics. If requesting major revision, please articulate the new experiments that are needed.

Reviewer #1: The authors adequately answered all my questions. I suggest that the manuscript can be accepted.

Reviewer #2: Congratulations to the authors on an excellent paper. Thank you for considering all suggestions made in my first review and taking the time to incorporate these in the manuscript.

Although I overlooked this on first read, the authors may want to consider referencing a paper by Williams et al 'Clinical studies of the effectiveness and safety of antivenoms, Toxicon 2018, PMID: 29746978' in the introduction that highlights the disparity in outcome reporting in clinical snakebite trials and offers a framework for reporting, albeit without the evidence generated in this systematic review.

**********

PLOS authors have the option to publish the peer review history of their article (what does this mean?). If published, this will include your full peer review and any attached files.

If you choose “no”, your identity will remain anonymous but your review may still be made public.

**Do you want your identity to be public for this peer review?** For information about this choice, including consent withdrawal, please see our Privacy Policy.

Reviewer #1: No

Reviewer #2: **Yes: **Thomas Lamb

# Editor report: Acceptance letter

**DOI:** 10.1371/journal.pntd.0009589.r004

**Authors:** Abdulrazaq G. Habib, Kempaiah Kemparaju

14 Jul 2021

Dear Dr. Abouyannis,

We are delighted to inform you that your manuscript, "Clinical outcomes and outcome measurement tools reported in randomised controlled trials of treatment for snakebite envenoming: a systematic review," has been formally accepted for publication in PLOS Neglected Tropical Diseases.

We have now passed your article onto the PLOS Production Department who will complete the rest of the publication process. All authors will receive a confirmation email upon publication.

The corresponding author will soon be receiving a typeset proof for review, to ensure errors have not been introduced during production. Please review the PDF proof of your manuscript carefully, as this is the last chance to correct any scientific or type-setting errors. Please note that major changes, or those which affect the scientific understanding of the work, will likely cause delays to the publication date of your manuscript. Note: Proofs for Front Matter articles (Editorial, Viewpoint, Symposium, Review, etc...) are generated on a different schedule and may not be made available as quickly.

Soon after your final files are uploaded, the early version of your manuscript will be published online unless you opted out of this process. The date of the early version will be your article's publication date. The final article will be published to the same URL, and all versions of the paper will be accessible to readers.

Thank you again for supporting open-access publishing; we are looking forward to publishing your work in PLOS Neglected Tropical Diseases. 

Best regards,

Shaden Kamhawi

co-Editor-in-Chief

PLOS Neglected Tropical Diseases

Paul Brindley

co-Editor-in-Chief

PLOS Neglected Tropical Diseases
